# Alpha taxonomy of the genus *Kessleria* Nowicki, 1864, revisited in light of DNA-barcoding (Lepidoptera, Yponomeutidae)

**DOI:** 10.3897/zookeys.503.9590

**Published:** 2015-05-11

**Authors:** Peter Huemer, Marko Mutanen

**Affiliations:** 1 Naturwissenschaftliche Sammlungen, Tiroler Landesmuseen Betriebgsges.m.b.H., Feldstr. 11a, A-6020 Innsbruck, Austria; 2 Department of Genetics and Physiology, University of Oulu, Oulu, Finland

**Keywords:** Lepidoptera, Yponomeutidae, *Kessleria*, new species, integrative taxonomy, DNA barcode, morphology, cryptic diversity, European mountains

## Abstract

The taxonomy of *Kessleria*, a highly specialized montane genus of Yponomeutidae with larval host restriction to Saxifragaceae and Celastraceae (Saxifraga spp. – subgenus Kessleria; *Saxifraga* spp. and Parnassia spp. – subgenus Hofmannia), is revised based on external morphology, genitalia and DNA barcodes. An integrative taxonomic approach supports the existence of 29 species in Europe (the two known species from Asia and North America are not treated herein). A full 658 bp fragment of COI was obtained from 135 specimens representing 24 species, a further seven sequences are >560 bp. Five new species are described: *Kessleria cottiensis* **sp. n.** (Prov. Torino, Italy; Dep. Hautes Alpes, France), *Kessleria dimorpha* **sp. n.** (Dep. Alpes-de-Haute-Provence, France), *Kessleria alpmaritimae* **sp. n.** (Dep. Alpes-Maritimes, France), *Kessleria apenninica* **sp. n.** (Prov. Rieti, Prov. L´Aquila, Italy), and *Kessleria orobiae* **sp. n.** (Prov. Bergamo, Italy).

## Introduction

The genus *Kessleria* Nowicki, 1864 is one of the striking examples of long underestimated or neglected diversity in the generally well known fauna of European Lepidoptera. This deficiency of knowledge is reflected in the fact that only 9 out of the 29 European species were described before 1960, and 18 species, or two-thirds of the fauna, after 1990 (Friese 1960, Huemer and Tarmann 1992, 1993). The genus is exceptional in Lepidoptera due to its highly specialized host-plant relationship with the Saxifragaceae, a habit shared e.g. by a section of the Curculionidae genus *Dichotrachelus* (Merigalli et al. 2013). Whereby the large majority of species is restricted to *Saxifraga*, two species feed on the herbaceous Celastraceae genus *Parnassia*, long considered as Saxifragaceae (The Angiosperm Phylogeny Group 2009). Through these host-plant relationships, *Kessleria* is a genus characteristic of mountain regions in the northern hemisphere, reaching high altitudes of about 3000 m in the European Alps and only exceptionally occurring below 600 m. In such alpine environments adult morphology may be adapted to rough climatic conditions with female brachyptery observed in several families of Lepidoptera (Sattler 1991), including minimum five species of *Kessleria*. Two major revisions (Friese 1960, Huemer and Tarmann 1992) are primarily based on external and internal morphology of adults, supplemented by ecological data. These authors established a stable and undisputed alpha taxonomy of *Kessleria*, which has been in use for the last two decades. Recently discovered suspected morphospecies led to the implementation of molecular methods as an additional tool in species delimitation. Sequences of the COI barcode region (Hebert et al. 2003, 2009) confirmed the species status of the vast majority of previously described taxa, and helped in resolving suspected cryptic species-complexes and in delimiting five species new to science.

## Material and methods

Extensive descriptions and diagnoses of previously described European species of *Kessleria* including keys to males and females, colour figures of adults, black-and-white figures of male and female genitalia, last abdominal segments, illustrations of wing venation and figures of larval habits and habitats have been published by Huemer and Tarmann (1992, 1993) and are not repeated here.

Our study was initially based on morphology of the extensive material published in detail by Huemer and Tarmann (1992), and about 100 additional specimens, with DNA barcode sequences as an additional tool for delimitation of cryptic species. Most of the material was set and dried according to standard practice, some were spread, and a few only pinned. Genitalia preparations followed standard techniques for microlepidoptera (Robinson 1976), adapted for *Kessleria* e.g. by the manual eversion of cornuti (Huemer and Tarmann 1992). Wing venation was not considered for new species descriptions as it proved irrelevant for alpha taxonomy in the genus in the earlier revision by Huemer and Tarmann (1992).

We tried to obtain DNA barcode sequences, a 658 base-pair long segment of the 5’ terminus of the mitochondrial COI gene (*cytochrome c oxidase 1*), from 150 specimens, three from LMK and ZMUO respectively, and 144 from TLMF. DNA samples (from a single dried leg) were prepared according to the accepted standards. Legs from 150 specimens of *Kessleria* were processed at the Canadian Centre for DNA Barcoding (CCDB, Biodiversity Institute of Ontario, University of Guelph) using their standard high-throughput protocol described in deWaard et al. (2008). Successfully sequenced voucher specimens are listed in Suppl. material 1 together with species names, sample-IDs, process-IDs, BINs, COI-5P sequence length, and trace counts. Sequences were submitted to GenBank during printing stage; GenBank accession numbers further details including complete voucher data and images can be accessed in the public dataset “Lepidoptera of Europe - *Kessleria*” http://dx.doi.org/10.5883/DS-LEAKE in the Barcode of Life Data Systems (BOLD; Ratnasingham and Hebert 2007). Degrees of intra- and interspecific variation in the DNA barcode fragment were calculated under the Kimura 2-parameter (K2P) model of nucleotide substitution using analytical tools in BOLD systems v3.0. (http://www.boldsystems.org). A neighbor-joining tree of DNA barcode data of European taxa was constructed using Mega 5 (Tamura et al. 2011) under the K2P model for nucleotide substitutions. In taxonomic delimitation, we applied principles of integrative taxonomy (Padial et al. 2010) and considered a barcode divergence of roughly 2% supported by at least one morphological character indicating species distinctiveness. We acknowledge that any threshold value of genetic distinctiveness is artificial and should not alone be used as indicating species status (cf. Collins and Cruickshank 2013), for which reason we considered 2% genetic difference associated with at least one morphological character indicating species integrity in the sense of e.g. General Lineage Species Concept (deQueiroz 1998) and Phylogenetic (diagnostic) Species Concept (Cracraft 1989), which both are applicable in delimiting also allopatric populations.

Photographs of the adults were taken with an Olympus SZX 10 binocular microscope and an Olympus E 3 digital camera, and processed using the software Helicon Focus 4.3 and Adobe Photoshop CS4 and Lightroom 2.3. Genitalia photographs were taken with an Olympus E1 Digital Camera from Olympus BH2 microscope.

Measurements were taken with a micrometer eyepiece.

### Abbreviations of institutional collections

BMNH Natural History Museum (British Museum, Natural History) London, United Kingdom

LMK Landesmuseum Kärnten, Klagenfurt, Austria

MNCN Museo Nacional de Ciencias Naturales, Madrid, Spain

MNHU Museum für Naturkunde der Humboldt Universität, Berlin, Germany

NHMV Naturhistorisches Museum, Vienna, Austria

SDEI Senckenberg Deutsches Entomologisches Institut, Müncheberg, Germany

SMNK Staatliches Museum für Naturkunde, Karlsruhe, Germany

TLMF Tiroler Landesmuseum Ferdinandeum, Innsbruck, Austria

ZMUO Zoological Museum, University of Oulu, Finland

ZMUC Zoological Museum, Natural History Museum of Denmark, University of Copenhagen, Copenhagen, Denmark

ZSM Zoologische Staatssammlung, München, Germany

## Results

The checklist of European *Kessleria* largely follows Huemer and Tarmann (1992). The proposed tentative structure into species groups is based on morphology and as far as available DNA barcode data, but a well-grounded phylogenetic analysis will require further data, particularly from nuclear markers.

### Checklist of European *Kessleria*

*Kessleria* Nowicki, 1864

Subgenus *Kessleria* Nowicki, 1864

*Kessleria alpicella*-group

*Kessleria alpicella* (Stainton, 1851)

= *Kessleria alpicella* (Herrich-Schäffer, 1855), Homonym

*Kessleria mixta* Huemer & Tarmann, 1992

*Kessleria alternans*-group

*Kessleria alternans* (Staudinger, 1871)

*Kessleria cottiensis* sp. n.

*Kessleria dimorpha* sp. n.

*Kessleria wehrlii* Huemer & Tarmann, 1992

*Kessleria alpmaritimae* sp. n.

*Kessleria petrobiella*-group

*Kessleria nivescens* Burmann, 1980

*Kessleria petrobiella* (Zeller, 1868)

*Kessleria albanica*-group

*Kessleria macedonica* Huemer & Tarmann, 1992

*Kessleria albanica* Friese, 1960

*Kessleria burmanni* Huemer & Tarmann, 1992

*Kessleria insubrica* Huemer & Tarmann, 1993

*Kessleria hauderi* Huemer & Tarmann, 1992

*Kessleria apenninica*-group

*Kessleria apenninica* sp. n.

*Kessleria diabolica* Huemer & Tarmann, 1992

*Kessleria brevicornuta* Huemer & Tarmann, 1992

*Kessleria pyrenaea* Friese, 1960

*Kessleria brachypterella* Huemer & Tarmann, 1992

*Kessleria zimmermanni*-group

*Kessleria zimmermanni* Nowicki, 1864

= *Kessleria tatrica* Friese, 1960

*Kessleria albomaculata* Huemer & Tarmann, 1992

*Kessleria caflischiella* (Frey, 1880)

*Kessleria albescens*-group

*Kessleria klimeschi* Huemer & Tarmann, 1992

*Kessleria helvetica* Huemer & Tarmann, 1992

*Kessleria inexpectata* Huemer & Tarmann, 1992

*Kessleria orobiae* sp. n.

*Kessleria albescens* (Rebel, 1899)

Subgenus *Hofmannia* Heinemann & Wocke, 1877

*Kessleria saxifragae* (Stainton, 1868)

*Kessleria fasciapennella* (Stainton, 1849)

= *Kessleria longipenella* Friese, 1960

### Molecular analysis

**Figure 1.**
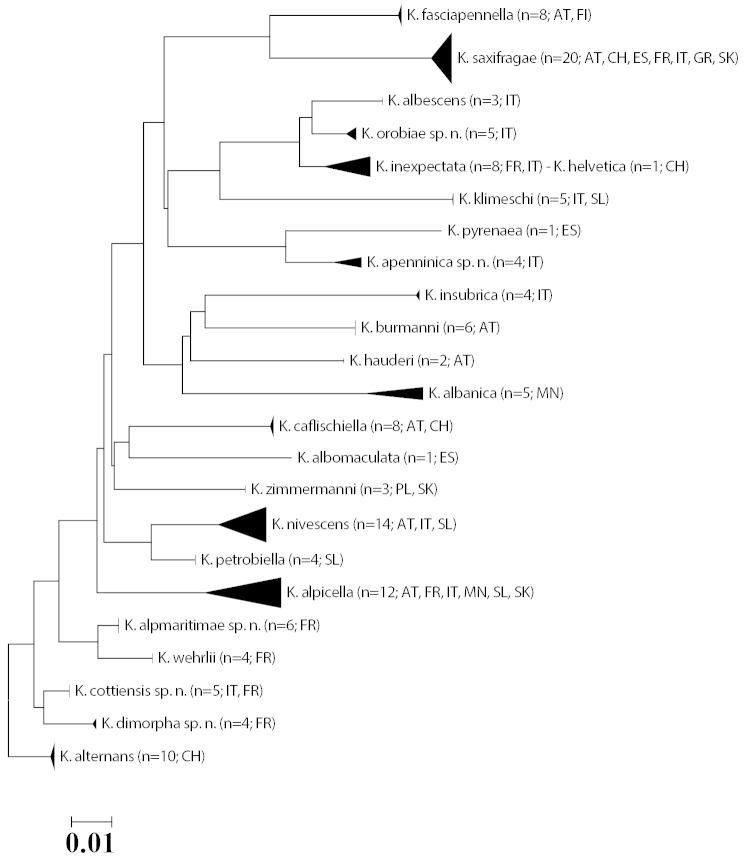
Neighbour-joining tree (Kimura 2 parameter, built with MEGA 5; cf. Tamura et al. 2011). Note: the scale bar only applies to internal branches between species. The width of the triangles represents the sample size, and the depth the relative genetic variation within the cluster (2× scale bar). Source: DNA Barcode data from BOLD (Barcode of Life Database, cf. Ratnasingham and Hebert 2007).

Sequencing resulted in a full barcode fragment of 658 bp for 135 specimens, covering 24 species. A further seven sequences that were longer than 560 bp were included in the analysis. A single short sequence of 307 bp was not considered, and sequencing failed for seven voucher specimens. Mean intraspecific divergence is 0.61%. It ranges from 0–4.27%, exceeding 2% only in three species, which, however, may include further cryptic diversity (e.g. *Kessleria alpicella*, *Kessleria albanica* and *Kessleria inexpectata*) and should be tested accordingly with more material (Table 1, Fig. 1). On the contrary, interspecific divergence in the genus is much higher with a mean divergence of 10.38% and maximum of 16.22%. Interspecific divergence to the nearest neighbour ranges from 1.86–9.29%, with the only exception being *Kessleria inexpectata* and *Kessleria helvetica*, which overlap in DNA barcode (Table 1, Fig. 1).

From sequence analysis of 20 *Kessleria* species based on at least three sequences, 17 species are delimited by a minimum of one to a maximum of 10 diagnostic characters whereas *Kessleria inexpectata*, *Kessleria cottiensis* and *Kessleria alpmaritimae* have no diagnostic character (Table 1).

**Table 1.** Intraspecific distance and interspecific divergence to the nearest neighbour in the genus *Kessleria*. Source: DNA Barcode data from BOLD (Barcode of Life Database, cf. Ratnasingham and Hebert 2007).

Species	# sequ	Mean intra	Max intra	Nearest neighbour	Nearest species	Nearest species	Diagnostic characters
*Kessleria albanica*	5	2.05	2.98	PHLAB1059-10	*Kessleria burmanni*	9.29	8
*Kessleria albescens*	3	0	0	PHLAD145-11	*Kessleria orobiae*	2.66	3
*Kessleria albomaculata*	1	N/A	N/A	PHLAD138-11	*Kessleria petrobiella*	6.76	-
*Kessleria alpicella*	12	1.52	4.27	PHLAD119-11	*Kessleria wehrlii*	6.9	6
*Kessleria alpmaritimae*	6	0	0	PHLAD119-11	*Kessleria wehrlii*	1.87	0
*Kessleria alternans*	10	0.12	0.31	PHLAD122-11	*Kessleria cottiensis*	2.65	3
*Kessleria apenninica*	4	1.06	1.71	PHLAI438-13	*Kessleria pyrenaea*	5.47	3
*Kessleria burmanni*	6	0	0	PHLAD140-11	*Kessleria hauderi*	7.61	6
*Kessleria caflischiella*	8	0.04	0.15	PHLAD118-11	*Kessleria alpmaritimae*	6.39	6
*Kessleria cottiensis*	5	0	0	PHLAB957-10	*Kessleria dimorpha*	1.86	0
*Kessleria dimorpha*	4	0.08	0.15	PHLAD122-11	*Kessleria cottiensis*	1.86	1
*Kessleria fasciapennella*	8	0.04	0.15	PHLAI063-12	*Kessleria saxifragae*	7.21	8
*Kessleria hauderi*	2	0	0	PHLAB1059-10	*Kessleria burmanni*	7.61	-
*Kessleria helvetica*	1	N/A	N/A	PHLAB1065-10	*Kessleria inexpectata*	0.31	-
*Kessleria inexpectata*	7	1.4	2.18	LASTS544-14	*Kessleria helvetica*	0.31	0
*Kessleria insubrica*	4	0.08	0.15	PHLAB1059-10	*Kessleria burmanni*	8.95	9
*Kessleria klimeschi*	5	0.06	0.15	PHLAB1065-10	*Kessleria inexpectata*	8.83	10
*Kessleria nivescens*	14	1.09	2.5	PHLAD138-11	*Kessleria petrobiella*	3.29	4
*Kessleria orobiae*	5	0.31	0.46	PHLAB1067-10	*Kessleria albescens*	2.66	1
*Kessleria petrobiella*	4	0	0	PHLAD132-11	*Kessleria nivescens*	3.29	1
*Kessleria pyrenaea*	1	N/A	N/A	PHLAB861-10	*Kessleria apenninica*	5.47	-
*Kessleria saxifragae*	20	0.44	1.29	LEFIB126-10	*Kessleria fasciapennella*	7.21	9
*Kessleria wehrlii*	4	0	0	PHLAD118-11	*Kessleria alpmaritimae*	1.87	1
*Kessleria zimmermanni*	5	0	0	PHLAD138-11	*Kessleria petrobiella*	5.73	6

## Taxonomy

### New species of *Kessleria*

#### *Kessleria alternans*-group

The *Kessleria alternans*-group is characterized by strong sexual dichroism and to a lesser extent dimorphism, with females being smaller and lighter, but not strongly brachypterous (Figs 2–11). The genitalia are characterized by the strong reticulate sculpture of the apical part of the phallus (Figs 12–21) and the ductus bursae, which is extended into the corpus bursae (Figs 22–26). Larval host-plants, as far as known, belong to the small-leaved *Saxifraga* spp., particularly the *Saxifraga oppositifolia*-complex, and to broad-leaved congeners such as *Saxifraga paniculata*. Five species belong to this group: *Kessleria alternans*, *Kessleria wehrlii* and the new taxa *Kessleria cottiensis*, *Kessleria dimorpha* and *Kessleria alpmaritimae*.

**Figures 2–7.**
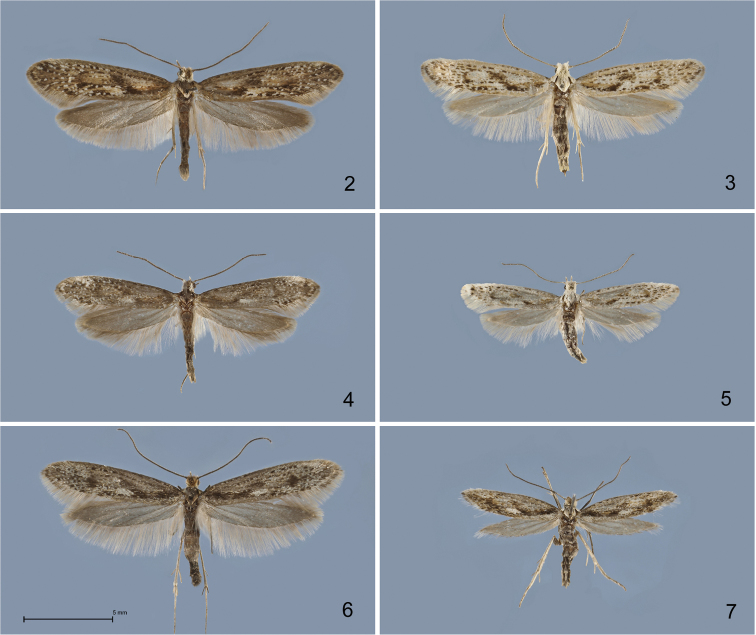
*Kessleria* adults in dorsal view. **2** *Kessleria alternans*, ♂, Switzerland, Graubünden, SE Sils-Maria, 1820–1870 m, 13.7.1989, leg. Huemer, Karsholt & Tarmann (TLMF) **3** *Kessleria alternans*, ♀, same data (TLMF) **4** *Kessleria cottiensis* sp. n., ♂, paratype, Italy, Prov. Torino, Alpi Cozie, V. delle Finestre, 1700 m, 27.7.1990, leg. Huemer & Tarmann (TLMF) **5** *Kessleria cottiensis* sp. n., ♀, paratype, same data (TLMF) **6** *Kessleria dimorpha* sp. n., ♂, paratype, France, Dep. Hautes-Alpes, Col Agnel, 2770 m, 4.8.2010, leg. Huemer (TLMF) **7** *Kessleria dimorpha* sp. n., ♀, paratype, same data (TLMF).

**Figures 8–11.**
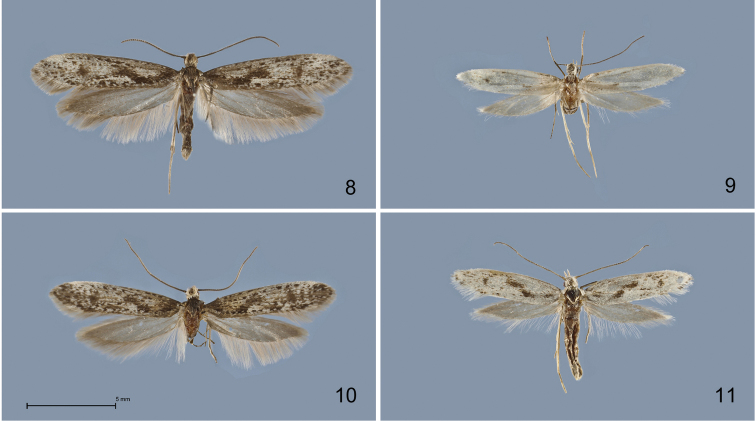
*Kessleria* adults in dorsal view. **8** *Kessleria wehrlii*, ♂, paratype, France, Dep. Alpes Maritimes, Mont Gelas Massiv, Mont Colomb W, 2450 m, 24.7.1990, leg. Huemer & Tarmann (DNA barcode ID TLMF Lep 01857) (TLMF) 9 *Kessleria wehrlii*, ♀, paratype, same data (TLMF) **10** *Kessleria alpmaritimae* sp. n., ♂, paratype, France, Dep. Alpes Maritimes, Marguareis W-Hang, Navela, 2100–2200 m, 18.–19.7.1991, leg. Huemer & Tarmann (TLMF) **11** *Kessleria alpmaritimae* sp. n., ♀, paratype, same data (DNA barcode ID TLMF Lep 01851) (TLMF).

**Figures 12–17.**
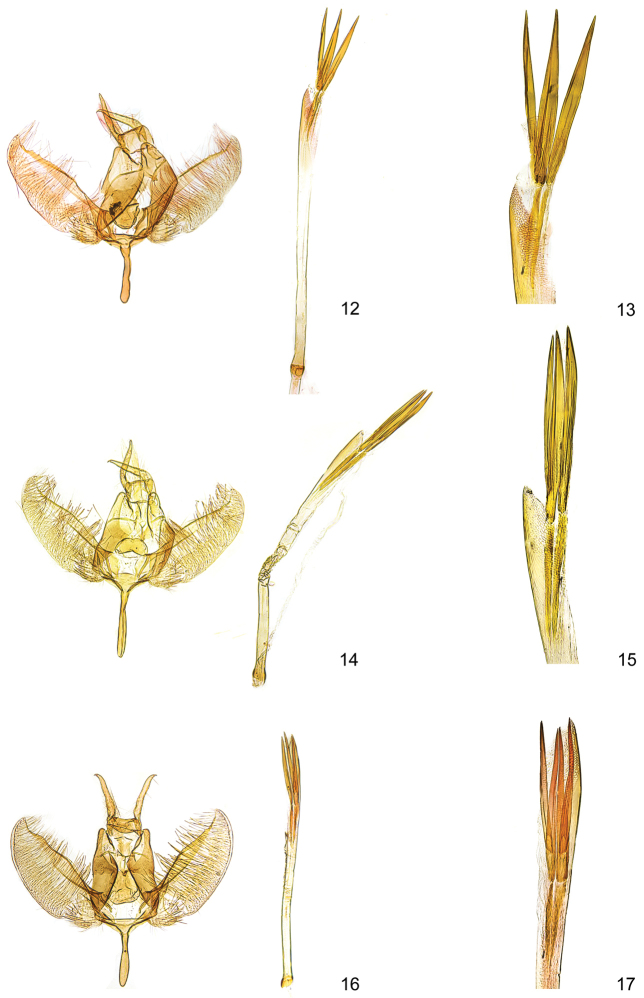
*Kessleria* male genitalia. **12** *Kessleria alternans*, Switzerland, Graubünden, SE Sils-Maria, 1820–1870 m, 13.7.1989, leg. Huemer, Karsholt & Tarmann, gen. slide YPO 22 (TLMF) **13** idem, distal part of phallus enlarged **14** *Kessleria cottiensis* sp. n., paratype, Italy, Prov. Torino, Alpi Cozie, V. delle Finestre, 1700 m, 27.7.1990, leg. Huemer & Tarmann, gen. slide YPO 66 (TLMF) **15** idem, distal part of phallus enlarged **16** *Kessleria dimorpha* sp. n., paratype, France, Dep. Hautes-Alpes, Col Agnel, 2770 m, 4.8.2010, leg. Huemer gen. slide YPO 149 (TLMF) **17** idem, holotype, gen. slide YPO 158, distal part of phallus enlarged.

**Figures 18–21.**
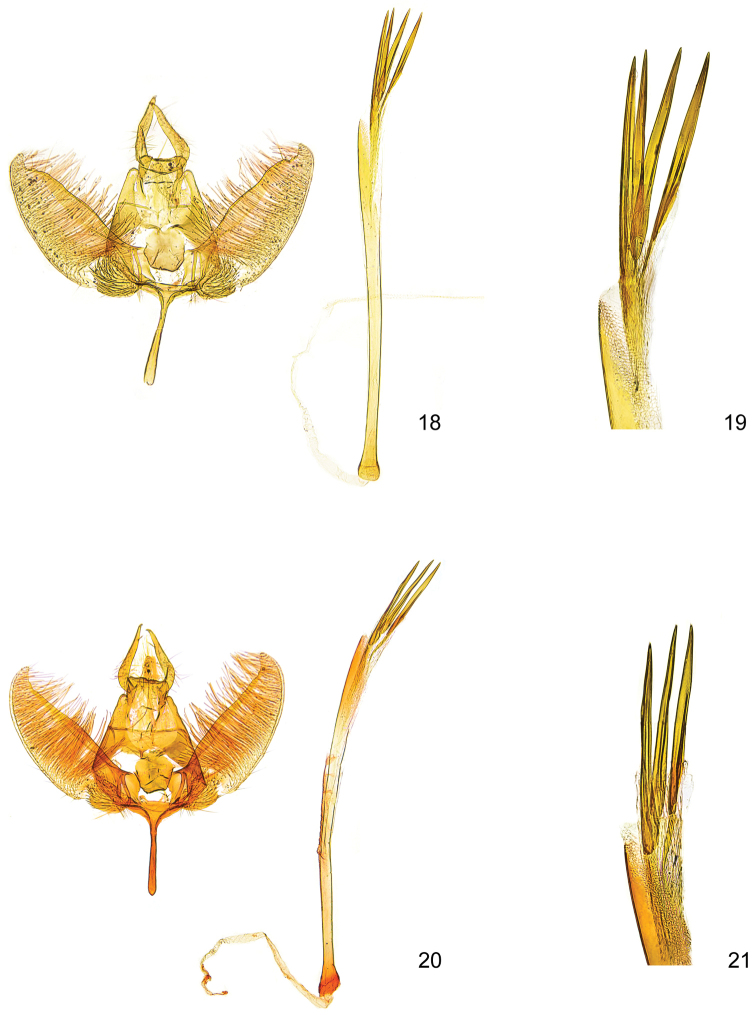
*Kessleria* male genitalia. **18** *Kessleria wehrlii*, paratype, France, Dep. Alpes Maritimes, Mont Gelas Massiv, Mont Colomb W, 2450 m, 24.7.1990, leg. Huemer & Tarmann (DNA barcode ID TLMF Lep 01857), gen. slide YPO 64 (TLMF) **19** idem, distal part of phallus enlarged **20** *Kessleria alpmaritimae* sp. n., paratype, France, Dep. Alpes Maritimes, Marguareis W-Hang, Navela, 2100–2200 m, 18.-19.7.1991, leg. Huemer & Tarmann, gen. slide YPO 55 (TLMF) **21** idem, distal part of phallus enlarged.

**Figures 22–23.**
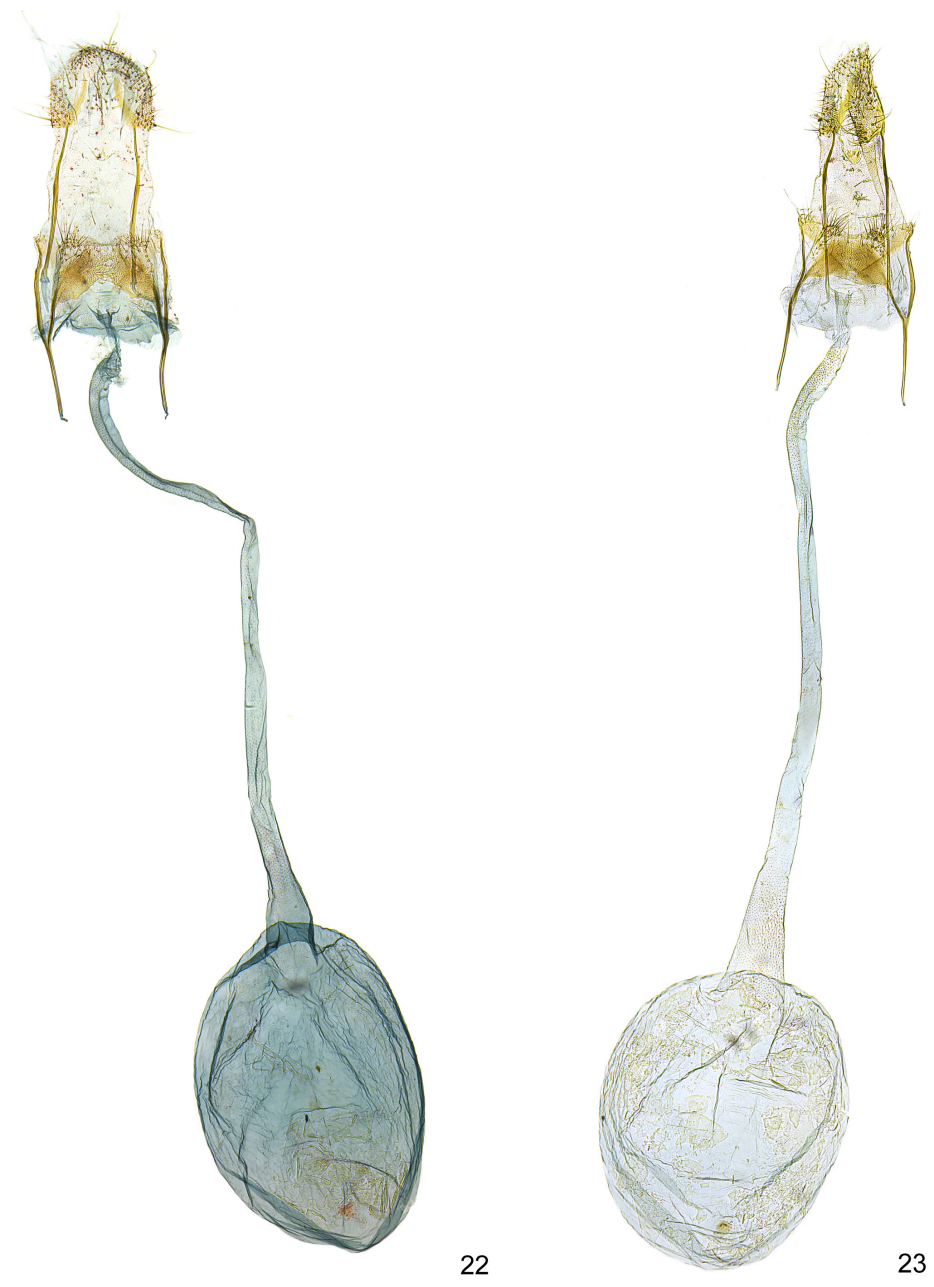
*Kessleria* female genitalia. **22** *Kessleria alternans*, Switzerland, Graubünden, SE Sils-Maria, 1820–1870 m, 13.7.1989, leg. Huemer, Karsholt & Tarmann, gen. slide YPO 6 (TLMF) **23** *Kessleria cottiensis* sp. n., paratype, Italy, Prov. Torino, Alpi Cozie, V. delle Finestre, 1700 m, 27.7.1990, leg. Huemer & Tarmann, gen. slide YPO 67 (TLMF).

**Figures 24–26.**
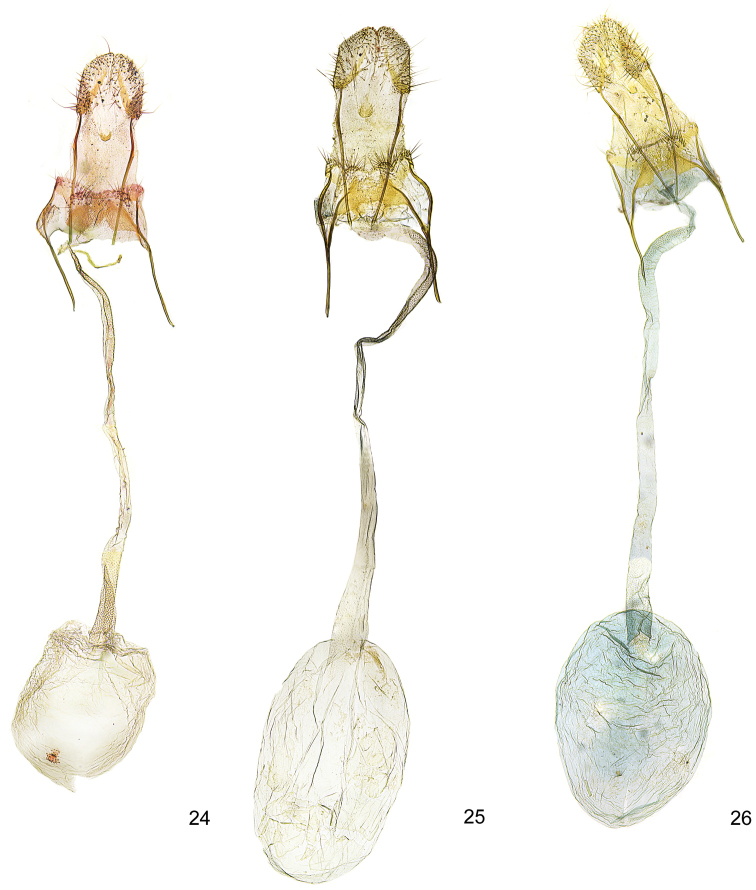
*Kessleria* female genitalia. **24** *Kessleria dimorpha* sp. n., paratype, France, Dep. Hautes-Alpes, Col Agnel, 2770 m, 4.8.2010, leg. Huemer, gen. slide YPO 159 (TLMF) **25** *Kessleria wehrlii*, paratype, France, Dep. Alpes Maritimes, Mont Gelas Massiv, Mont Colomb W, 2450 m, 24.7.1990, leg. Huemer & Tarmann, gen. slide YPO 69 (TLMF) **26** *Kessleria alpmaritimae* sp. n., paratype, France, Dep. Alpes Maritimes, Marguareis W-Hang, Navela, 2100–2200 m, 18.-19.7.1991, leg. Huemer & Tarmann (TLMF).

**Figures 27–31.**
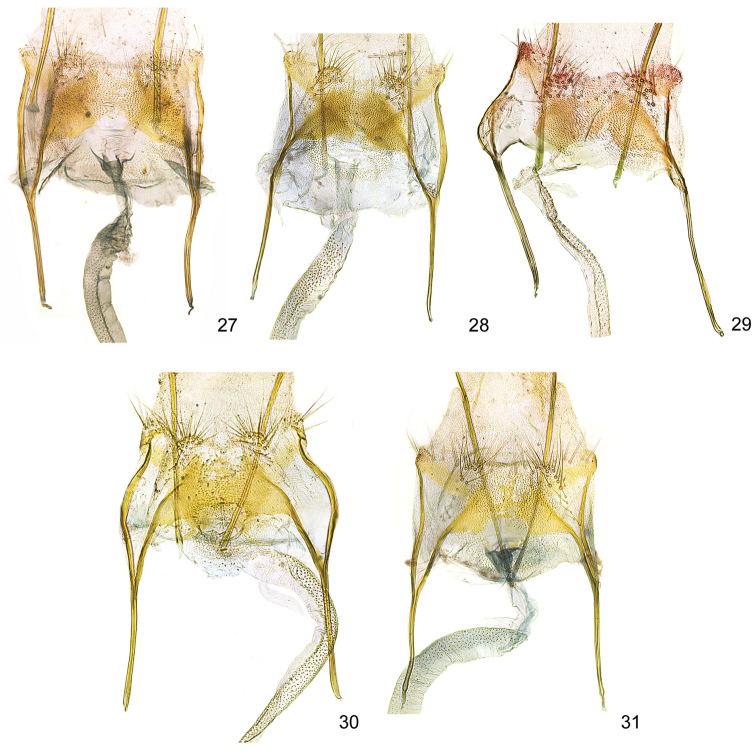
*Kessleria* female genitalia, details of VIII abdominal segment enlarged. **27** *Kessleria alternans*, Switzerland, Graubünden, SE Sils-Maria, 1820–1870 m, 13.7.1989, leg. Huemer, Karsholt & Tarmann, gen. slide YPO 6 (TLMF) **28** *Kessleria cottiensis* sp. n., paratype, Italy, Prov. Torino, Alpi Cozie, V. delle Finestre, 1700 m, 27.7.1990, leg. Huemer & Tarmann, gen. slide YPO 67 (TLMF) **29** *Kessleria dimorpha* sp. n., paratype, France, Dep. Hautes-Alpes, Col Agnel, 2770 m, 4.8.2010, leg. Huemer, gen. slide YPO 159 (TLMF) **30** *Kessleria wehrlii*, paratype, France, Dep. Alpes Maritimes, Mont Gelas Massiv, Mont Colomb W, 2450 m, 24.7.1990, leg. Huemer & Tarmann, gen. slide YPO 69 (TLMF) **31** *Kessleria alpmaritimae* sp. n., paratype, France, Dep. Alpes Maritimes, Marguareis W-Hang, Navela, 2100–2200 m, 18.–19.7.1991, leg. Huemer & Tarmann (TLMF).

**Figure 32.**
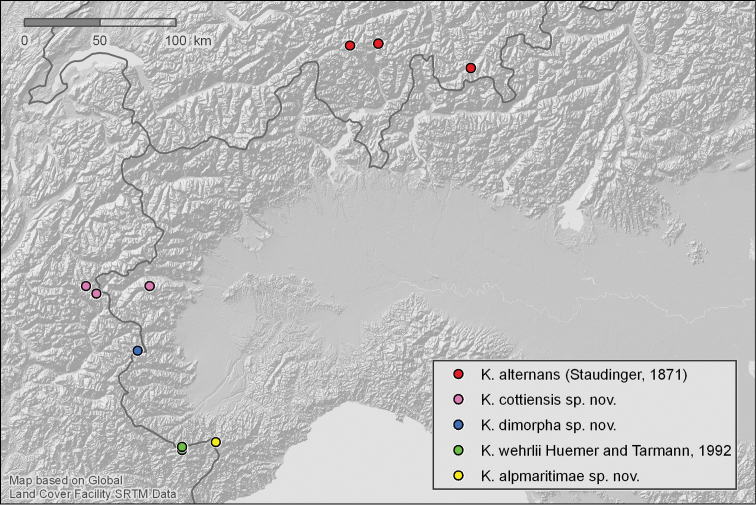
Distribution pattern of the *Kessleria alternans*-group from examined material.

**Figures 33–34.**
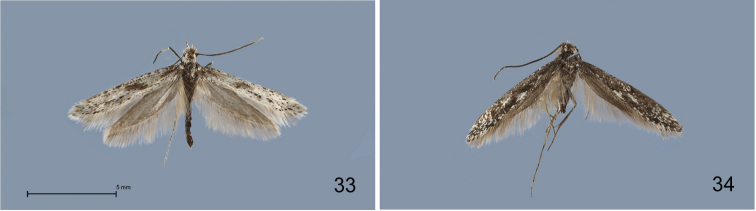
*Kessleria* adults in dorsal view. **33** *Kessleria apenninica* sp. n., ♂, holotype, Italy, L´Aquila, NP Gran Sasso, ex Miniera di Lignite, 1750 m, 14.-15.7.2010, leg. Huemer (DNA barcode ID TLMF Lep 01663) (TLMF) **34** *Kessleria pyrenaea*, ♂, Spain, Aragon, Parzan (Bielsa) env. Pico de la Rubinera, 2700–3005 m, 10.-11.7.2010, leg. Cesanek (DNA barcode ID TLMF Lep 08933) (TLMF) (coll. Tokár).

**Figures 35–38.**
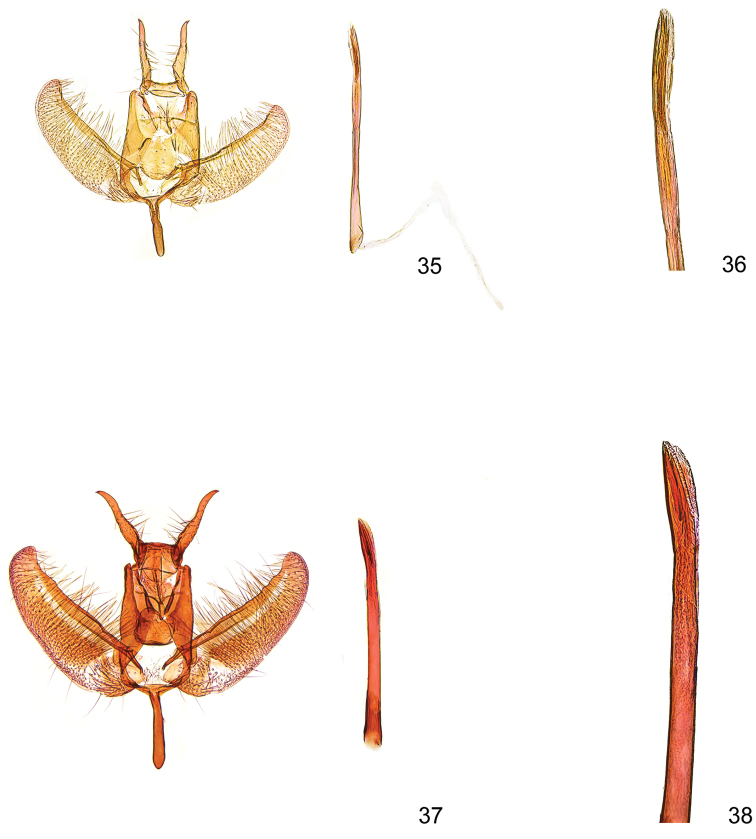
*Kessleria* male genitalia. **35** *Kessleria apenninica* sp. n., ♂, holotype, Italy, L´Aquila, NP Gran Sasso, ex Miniera di Lignite, 1750 m, 14.-15.7.2010, leg. Huemer gen. slide YPO 147 (DNA barcode ID TLMF Lep 01663) (TLMF) **36** idem, distal part of phallus enlarged **37** *Kessleria pyrenaea*, ♂, Spain, Aragon, Parzan (Bielsa) env. Pico de la Rubinera, 2700–3005 m, 10.–11.7.2010, leg. Cesanek, gen. slide 15/1391 P.Huemer (DNA barcode ID TLMF Lep 08933) (coll. Tokár) **38** idem, distal part of phallus enlarged.

##### Kessleria cottiensis sp. n.

Taxon classificationAnimaliaLepidopteraYponomeutidae

http://zoobank.org/21EBBA7D-08EE-4713-B958-F67F00B0CEE5

###### Type material.

**Holotype.** ♂, „ITALIA, Prov. Torino Alpi Cozie, 2150 m Colle delle Finestre 27.7.1990 leg. Huemer & Tarmann“ „YPO 58 ♂ P. Huemer“ (TLMF).

**Paratypes. Italy:** 13 ♂, 7 ♀, same data, genitalia slides YPO 59 ♂ P. Huemer, YPO 77 ♂ P. Huemer, DNA barcode IDs TLMF Lep 03106, TLMF Lep 03107, TLMF Lep 03108 (TLMF); 3 ♂, 6 ♀, same data, but V. delle Finestre, 1700 m, genitalia slides YPO 66 ♂ P. Huemer, YPO 67 ♀ P. Huemer (TLMF). **France:** 1 ♂, Dep. Hautes-Alpes, Nevache, 1950m, 31.7.2001, leg. Nel, genitalia slide 12937 J. Nel, DNA barcode ID TLMF Lep 03144 (TLMF); 1 ♂, Dep. Hautes-Alpes, Vallee de la Claree, 2000 m, 3.7.2002, leg. Nel, genitalia slide 14644 J. Nel, DNA barcode ID TLMF Lep 03142 (TLMF).

###### Diagnosis.

*Kessleria cottiensis* resembles other taxa of the *Kessleria alternans*-group in wing markings and colour (Figs 2–11), but the male differs in the on average smallest forewing length of 7.59 mm (n=14) *vs.* 8.75 mm (n=13) in *Kessleria wehrlii*, 8.55 mm (n=26) in *Kessleria alternans*, 8.05 mm (n=6) in *Kessleria alpmaritimae* and 8.25 mm (n=6) in *Kessleria dimorpha*. Compared to *Kessleria wehrlii* and *Kessleria alpmaritimae*, the whitish suffusion of the forewing is largely reduced. The ochre-brown markings, which are well present in *Kessleria alternans*, are almost completely absent. The female of *Kessleria cottiensis* is also distinctly smaller than *Kessleria alternans*, with a forewing length of 6.13 mm (n=8) on average *vs.* 7.58 mm (n=11), whereas females of *Kessleria cottiensis* and *Kessleria alpmaritimae* are indistinguishable. *Kessleria wehrlii* is insufficiently known from only a single worn female specimen. Compared to the genetically nearest neighbour *Kessleria dimorpha*, which is similar in forewing length (6.0 mm, n=4), the hindwings are slightly less reduced and the ground colour of the forewing is much lighter. Diagnostic characters in genitalia are generally weak. The male genitalia differ from *Kessleria alpmaritimae* by the medially strongly widened valva, from *Kessleria alternans* by the more slender cornuti, from *Kessleria wehrlii* by three instead of four cornuti, and from the nearest neighbour *Kessleria dimorpha* by the distinctly longer phallus (1.65–1.70 mm *vs.* 1.32–1.36 mm) (Figs 12–21). The female genitalia show no diagnostic characters to related species of the *Kessleria alternans*-group (Figs 22–31).

###### Description.

**Male** (Fig. 4). Head covered with erected whitish hair-like scales; antennae dark grey-brown, indistinctly lighter ringed; thorax and tegulae dark grey-brown. Forewing length 7.0–8.8 mm (Ø 7.59 mm; n=14); ground colour dark grey-brown, mottled with whitish scales, particularly in distal half, forming indistinct patches in fold and on costa at about 4/5; irregular black dots on veins and few brown scales in medial part of wing; oblique blackish fascia at about 1/3 to 1/2 indistinct; termen mixed whitish-grey, dark grey-brown in apical part; fringes light grey, darker in apical area. Hindwing dark grey, fringes with dark grey base, distal part light grey.

**Female** (Fig. 5). Head covered with erected whitish hair-like scales; antennae grey-brown, distinctly ringed whitish; thorax and tegulae whitish. Forewing length 5.8–6.3 mm (Ø 6.13 mm; n=8); ground colour whitish, mottled with black scales, particularly along veins and in tornal part, patches of brown scales in medial part of wing from base to end of cell; oblique blackish fascia at about 1/3 to 1/2 indistinct, separated into larger dash-like patch and reduced dot; termen whitish with some dark grey-brown mottling in apical part; fringes greyish-white, darker in apical area.

**Male genitalia** (Figs 14–15). Socii long and slender, with apical spine; anterior margin of tegumen with medial process; gnathos broadly tongue-shaped, smooth; valva moderately slender, length 0.66–0.70 mm, medially distinctly widened, maximum width of about 0.24–0.26 mm, densely covered with long hairs in medial part and short setae on ventromedial margin, ventromedial part weakly convex, distal part slender with ventrally convex and dorsally projected apex, costa strongly sclerotized with some distal dentation; sacculus oval, weakly confined, densely covered with strong setae; saccus sizeable in length, about 0.38–0.42 mm, stout, about same width throughout, apex rounded; phallus ca. 1.65–1.70 mm long and slender, straight, apically with distinct reticulate sculpture, uneverted vesica with ca. 0.76–0.80 mm long sclerotized part, three prominent needle-shaped, one single, the other basally connected, cornuti of about 0.46–0.51 mm in length.

**Female genitalia** (Figs 23, 28). Genitalia ca. 4.9 mm in length; papilla analis large, densely covered with long setae; apophysis posterior rod like, ca. 0.70 mm, about length of apophysis anterior; apophysis anterior rod like; posterior part bifurcated with straight dorsal and inwardly curved ventral branch; lamella postvaginalis with large sclerotized mediolateral patches, covered with microtrichia, medial area less sclerotized, posteriolateral part with hump, covered with some long setae; ostium bursae membranous; antrum weakly sclerotized, funnel-shaped; ductus bursae very long, about 2.8 mm, from entrance of ductus seminalis to transition into corpus bursae covered with finely granulous sculpture, particularly in posterior and anterior part, ductus bursae extended into posterior part of corpus bursae, entrance to corpus bursae weakly widened; corpus bursae well delimited, about 1.2 mm in length, ovoid, with small plate-like signum.

###### Molecular data.

The average intraspecific divergence of the barcode region is 0.0% (n=5). The minimum distance to the nearest neighbour *Kessleria dimorpha* is 1.86%, whereas the minimum divergence to *Kessleria alternans*, *Kessleria alpmaritimae* and *Kessleria wehrlii* ranges from 2.65% and 2.98% to 3.63%, respectively.

###### Etymology.

The species name refers to the type locality in the Cottian Alps (Alpi Cozie, Alpes cottiennes).

###### Distribution

(Fig. 32). Only known from a small area in the southwestern Alps (Cottian Alps) of Italy and France. An alleged *Kessleria alternans* from the Graian Alps (Huemer and Tarmann 1992) likely refers to *Kessleria cottiensis*, but the specimen in question could not be re-examined.

###### Ecology.

Host-plant and early stages unknown. The adults were collected in late July. The flight period can most likely be further prolonged, depending on snow coverage and elevation. A specimen collected earlier during the summer, on June 9^th^, by Jäckh in Valle delle Finestre (Huemer and Tarmann 1992) probably belongs to *Kessleria cottiensis*. The adults were collected during the day, flying freely in the morning hours and flushed out from their resting places with a bee-smoker. The species occurs in alpine grassland interspersed with calcareous rocks. Vertical distribution: from about 1700 m to 2150 m.

###### Remarks.

*Kessleria cottiensis* described here was already suspected to be distinctive from *Kessleria alternans* by Huemer and Tarmann (1992), who illustrated adults (Figs 6–7) and cornuti of male genitalia (Fig. 105).

##### Kessleria dimorpha sp. n.

Taxon classificationAnimaliaLepidopteraYponomeutidae

http://zoobank.org/B77D97D9-D8B3-4829-A8B6-4A7C13D5434A

###### Type material.

**Holotype.** ♂, „Frankreich Dep. Hautes-Alpes Col Agnel, 2770 m 6°59'02"E, 44°41'10"N 4.8.2010, leg. Huemer TLMF 2011-010“ „BC TLMF Lep 01756“ „YPO 158 ♂ P. Huemer” (TLMF).

**Paratypes. France:** 7 ♂, 5 ♀, same data, genitalia slides YPO 149 ♂ P. Huemer, YPO 159 ♀ P. Huemer, DNA barcode IDs TLMF Lep 01757, TLMF Lep 01758, TLMF Lep 01759 (TLMF); 4 ♂, 1 ♀, same data, leg. Wieser (LMK).

###### Diagnosis.

*Kessleria dimorpha* resembles other taxa of the *Kessleria alternans*-group in wing markings and colour (Figs 2–11), but the male differs from the genetically nearest neighbour *Kessleria cottiensis* by the on average distinctly larger forewing length of 8.25 mm (n=6) *vs.* 7.59 mm (n=14). Larger species are *Kessleria wehrlii* with forewing length 8.75 mm (n=13) and *Kessleria alternans* with 8.55 mm (n=26), whereas *Kessleria alpmaritimae* with 8.05 mm (n=6) is of similar size. Furthermore, *Kessleria wehrlii* and *Kessleria alpmaritimae* have a much more prominent whitish suffusion on the forewing, whereas the ochre-brown markings of *Kessleria dimorpha* rather resemble *Kessleria alternans*. The female of *Kessleria dimorpha* reflects a tendency to reinforced brachyptery and is distinctly smaller than *Kessleria alternans* with a forewing length of only 6.0 mm (n=4) on average *vs.* 7.58 mm (n=11), whereas females of *Kessleria cottiensis* and *Kessleria alpmaritimae* are strongly suffused with whitish scales. *Kessleria wehrlii* is insufficiently known from only a single worn female specimen. The male genitalia differ from all other taxa of the *Kessleria alternans*-group by the distinctly shorter phallus with <1.40 mm *vs.* a minimum of 1.50 mm in other species (Figs 12–21). The female genitalia show no diagnostic characters to related species of the *Kessleria alternans*-group (Figs 22–31).

###### Description.

Male (Fig. 6). Head covered with ochre-brown hair-like scales; antennae almost unicolorous dark grey-brown; thorax and tegulae mixed dark grey-brown and ochre-brown. Forewing length 8.0–8.4 mm (Ø 8.25 mm; n=6); ground colour dark grey, intensively mottled with light grey, ochre-brown and whitish scales, white medial patch in fold; black dots particularly on costal and subcostal veins; black patch near base and at end of cell, oblique blackish fascia at about 1/3 to 1/2 reduced to large patch in fold; termen mixed dark and light grey; fringes basally dark grey, distal part whitish-grey, darker in apical area. Hindwing dark grey, fringes with dark grey base, distal part whitish-grey.

**Female** (Fig. 7). Head covered with erected whitish hair-like scales; antennae grey-brown, indistinctly lighter ringed; thorax and tegulae whitish. Forewing length 6.0 mm (Ø 6.0 mm; n=4); ground colour whitish, mottled with dark grey and black, particularly along fold and in tornal part, few black dots along costal and subcostal vein, small patches of ochre-brown scales in medial part of wing particularly in fold and at end of cell; oblique blackish fascia at about 1/3 to 1/2 indistinct, separated into larger dash-like patch and reduced dot; termen mixed whitish and dark grey; fringes whitish-grey, with dark grey basal part near apex. Hindwing grey, fringes whitish-grey with darker basal part.

**Male genitalia** (Figs 16–17). Socii long and slender, with apical spine; anterior margin of tegumen with medial process; gnathos broadly tongue-shaped, smooth; valva moderately slender, length 0.71–0.72 mm, medially weakly widened, maximum width of about 0.26–0.28 mm, densely covered with long hairs in medial part and short setae on ventromedial margin, ventromedial part weakly convex, distal part moderately slender with ventrally convex and dorsally projected apex, costa strongly sclerotized with weak distal dentation; sacculus oval, weakly confined, densely covered with strong setae; saccus short, about 0.32 mm, stout, about same width throughout, apex rounded; phallus ca. 1.32–1.36 mm long and slender, straight, apically with distinct reticulate sculpture, uneverted vesica with ca. 0.58–0.60 mm long sclerotized part, three to four prominent needle-shaped cornuti of about 0.38–0.40 mm in length.

**Female genitalia** (Figs 24, 29). Genitalia ca. 4.9 mm in length; papilla analis large, densely covered with long setae; apophysis posterior rod like, ca. 0.72 mm, about length of apophysis anterior; apophysis anterior rod like; posterior part bifurcated with straight dorsal and inwardly curved ventral branch; lamella postvaginalis with large sclerotized mediolateral patches, covered with microtrichia, medial area less sclerotize, posterolateral part with hump, covered with some long setae; ostium bursae membranous; antrum weakly sclerotized, funnel-shaped; ductus bursae long, ca. 2.3 mm, posterior part from entrance of ductus seminalis anterior and anterior part covered with finely granulous sculpture, medial part with weak and hardly discernible sculpture, ductus bursae extended into posterior part of corpus bursae, entrance to corpus bursae weakly widened; corpus bursae well delimited, about 1.4 mm in length, ovoid, with small plate-like signum.

###### Molecular data.

The average intraspecific divergence of the barcode region is low with 0.08%, ranging from a minimum of 0% to a maximum of 0.15% (n=4). The minimum distance to the nearest neighbour *Kessleria cottiensis* is 1.86%, whereas the minimum divergence to *Kessleria alternans*, *Kessleria alpmaritimae* and *Kessleria wehrlii* ranges from 3.15% and 3.64% to 4.3%, respectively.

###### Etymology.

The species name refers to the remarkable sexual dimorphism.

###### Distribution

(Fig. 32). Only known from the type locality, the French side of Col Agnel (Cottian Alps), close to the Italian border.

###### Bionomics.

Host-plant and early stages unknown. Based on the type locality, the host-plant is most likely Saxifraga cf. oppositifolia. The adults have been collected in early August during the early morning hours from about 7–10a.m. at low temperatures between 2–5 °C. Males were flying actively during this period in search for females. Both sexes were later found in copula, often sitting on cushions of their suspected host-plant. A single female was found at light, attracted from its nearby habitat and crawling upwards to the light tower, but unable to fly actively. From personal observations of PH, it is likely that the slightly reinforced brachyptery of *Kessleria dimorpha* is combined with flightlessness. The species occurs in rocky habitat on siliceous soil. Vertical distribution: about 2800 m.

###### Remark.

Fringes of the examined females seem partially lost and thus may lead to a biased impression of the extent of wing reduction.

##### Kessleria alpmaritimae sp. n.

Taxon classificationAnimaliaLepidopteraYponomeutidae

http://zoobank.org/03637C4C-2AA3-489F-948E-A14CB72DD121

###### Type material.

**Holotype.** ♂, „FRANKREICH Dep. Alpes Maritimes Marguareis W-Hang Navela 2100–2200 m 21.–23.7.1990“„leg. Huemer, Tarmann“„YPO 79 ♂ P. Huemer“ (TLMF).

**Paratypes. France:** 9 ♂, 5 ♀, same data, genitalia slide YPO 55 ♂ P. Huemer (TLMF); 7 ♂, 2 ♀, same data, but 18.-19.7.1991, DNA barcode IDs TLMF Lep 01850, TLMF Lep 01851, TLMF Lep 03100, TLMF Lep 03101, TLMF Lep 03102, TLMF Lep 03103 (TLMF); 3 ♂, 5 ♀, same data, but 23.7.1990 (TLMF); 1 ♂, same data, but Punta Marguareis, 2450–2650 m, 23.7.1990 (TLMF).

###### Diagnosis.

*Kessleria alpmaritimae* resembles other taxa of *Kessleria alternans*-group in wing markings and colour (Figs 2–11), but the male with average forewing length of only 8.05 mm (n=6) is distinctly smaller than *Kessleria wehrlii* with 8.75 mm (n=13) and *Kessleria alternans* with 8.55 mm (n=26) and larger than *Kessleria cottiensis* with only 7.59 mm (n=14). *Kessleria dimorpha* with an average forewing length of 8.25 mm (n=6) is similar in size, but clearly differs by the largely reduced whitish suffusion of the forewing, a character stage which also applies to *Kessleria cottiensis* and *Kessleria alternans*, whereas *Kessleria wehrlii* is intensely mottled whitish. The female with forewing length of only 6.06 mm (n=3) is distinctly smaller than that of *Kessleria alternans* with 7.58 mm (n=11), but hardly separable from other species in size. Compared to the genetically nearest neighbour *Kessleria dimorpha*, the hindwings are less reduced and the ground colour of the forewing is much lighter. The female of *Kessleria wehrlii* is insufficiently described due to limited material, and the females of *Kessleria cottiensis* and *Kessleria alpmaritimae* are indistinguishable. The male genitalia differ from *Kessleria cottiensis* by the medially weakly widened valva, from *Kessleria alternans* by the more slender cornuti, from the nearest neighbour *Kessleria wehrlii* by three instead of four cornuti, and from *Kessleria dimorpha* by the distinctly longer phallus (1.52–1.58 mm *vs.* 1.32–1.36 mm) (Figs 12–21).

The female genitalia show no diagnostic characters to related species of the *Kessleria alternans*-group (Figs 22–31).

###### Description.

**Male** (Fig. 10). Head covered with erected whitish hair-like scales; antennae dark grey-brown, indistinctly lighter ringed; thorax and tegulae dark grey-brown. Forewing length 7.0–8.5 mm (Ø 8.05 mm; n=6); ground colour blackish to dark grey-brown, intensely mottled with whitish scales, particularly from basal fifth to 4/5; veins with distinct black dots, particularly along costa, subcosta, cubital and anal veins, medial and radial veins with indistinct brown lines; oblique blackish fascia at about 1/3 to 1/2 indistinct; termen dark grey-brown; fringes light grey, with slightly darker base and indistinct fringe linge. Hindwing dark grey, fringes light greyish-white with slightly darker base.

**Female** (Fig. 11). Head covered with erected whitish hair-like scales; antennae grey-brown, indistinctly ringed whitish; thorax and tegulae whitish. Forewing length 5.9–6.3 mm (Ø 6.06 mm; n=3); ground colour whitish, mottled with few black scales, particularly along veins, patches of black scales near base and at distal end of cell; oblique blackish fascia at about 1/3 to 1/2 distinct, separated into two dashes; termen whitish with some blackish dots in apical part; fringes white. Hindwing grey, fringes whitish-grey.

**Male genitalia** (Figs 20–21). Socii long and slender, with apical spine; anterior margin of tegumen with medial process; gnathos broadly tongue-shaped, smooth; valva moderately slender, length 0.72–0.76 mm, medially weakly widened, maximum width of about 0.26–0.27 mm, densely covered with long hairs in medial part and short setae on ventromedial margin, ventromedial part weakly convex, distal part slender with ventrally convex and dorsally projected apex, costa strongly sclerotized with weak distal dentation; sacculus oval, weakly confined, densely covered with strong setae; saccus short, about 0.32–0.33 mm, stout, about same width throughout, apex rounded; phallus ca. 1.52–1.58 mm long and slender, straight, apically with distinct reticulate sculpture, uneverted vesica with ca. 0.62–0.66 mm long sclerotized part, three prominent needle-shaped, one single, the other basally connected, cornuti of about 0.42–0.44 mm in length.

**Female genitalia** (Figs 26, 31). Genitalia ca. 4.9 mm in length; papilla analis large, densely covered with long setae; apophysis posterior rod like, ca. 0.76 mm, about length of apophysis anterior; apophysis anterior rod like; posterior part bifurcated with straight dorsal and inwardly curved ventral branch; lamella postvaginalis with large sclerotized mediolateral patches, covered with microtrichia, medial area less sclerotized, posterolateral part with hump, covered with some long setae; ostium bursae membranous; antrum weakly sclerotized, funnel-shaped; ductus bursae very long, ca. 2.4 mm, from entrance of ductus seminalis to transition into corpus bursae covered with finely granulous sculpture, particularly in posterior and anterior part, ductus bursae extended into posterior part of corpus bursae, entrance to corpus bursae weakly widened; corpus bursae well delimited, about 1.2 mm in length, ovoid, with largely reduced plate-like signum.

###### Molecular data.

The average intraspecific divergence of the barcode region is 0.0% (n=6). The minimum distance to the nearest neighbour *Kessleria wehrlii* is 1.87%, whereas the minimum divergence to *Kessleria cottiensis*, *Kessleria dimorpha* and *Kessleria alternans* ranges from 2.98% and 3.64% to 3.75%, respectively.

###### Etymology.

The species name is a made-up word which refers to the area of the type locality, the Alpes Maritimes.

###### Distribution

(Fig. 32). Only known from the type locality, the Marguareis Massif, in the French Alpes Maritimes.

###### Ecology.

Host-plant and early stages unknown. The adults have been collected in the last third of July during the day, flying freely in the morning hours and flushed out from their resting places with a bee-smoker. The species occurs in rocky habitat on calcareous soil. Vertical distribution: from about 2100 m to 2650 m.

###### Remarks.

*Kessleria alpmaritimae* described here was already suspected to be distinctive from *Kessleria alternans* by Huemer and Tarmann (1992), who illustrated adults (Figs 8–9).

#### *Kessleria apenninica*-group

The *Kessleria apenninica*-group s.str. only includes the new species *Kessleria apenninica* which is characterized e.g. by slender forewings. From characters of the male genitalia, such as the short cornuti, closer relatives are likely to be the Iberian *Kessleria diabolica*, *Kessleria brevicornuta*, *Kessleria brachypterella* and *Kessleria pyrenaea*, which all differ in adult morphology (see Figs 33–38, and Huemer and Tarmann 1992). These species together with *Kessleria apenninica* may form a larger species-group, but at present material is scarce and supporting molecular data are lacking. The nearest neighbour of the new species with a full DNA barcode is tentatively attached to *Kessleria pyrenaea* and considered for the differential diagnosis.

##### Kessleria apenninica sp. n.

Taxon classificationAnimaliaLepidopteraYponomeutidae

http://zoobank.org/F2B3CFD4-6A60-414F-9CB3-DC7E54424C45

###### Type material.

**Holotype.** ♂, „Italia Prov. Rieti Monte Terminillo 13°00,6'E, 42°29,0'N 1730–1780 m, 16.7.2010 leg. Huemer TLMF 2010-020“ „YPO 147 ♂ P. Huemer“ „TLMF Lep 01662“ (TLMF).

**Paratypes. Italy:** 1 ♂, same data, DNA barcode ID TLMF Lep 01661 (TLMF); 2 ♂, Prov. L´Aquila, NP Gran Sasso, ex Miniera di Lignite, 13°42,8'E, 42°25,6'N, 1750 m, 14.-15.7.2010, leg. Huemer, genitalia slide YPO 148 ♂ P. Huemer, DNA barcode IDs TLMF Lep 01663, TLMF Lep 01664 (TLMF).

###### Diagnosis.

*Kessleria apenninica* is characterized by unusually slender forewings and a pure white colour with black pattern. Species from the *Kessleria apenninica*-group are externally unmistakably distinguishable from one another both by wing pattern and colour (Figs 33–34, and Huemer and Tarmann 1992), but the genitalia of males are similar (Figs 35–38). However, in *Kessleria apenninica* the saccus is distinctly shorter than in all other species with 0.23 *vs.* minimum 0.29 mm.

###### Description.

**Male** (Fig. 33). Head covered with whitish hair-like scales; antennae almost unicolorous dark grey with light grey apex; thorax and tegulae mixed dark grey and whitish. Forewing length 5.8–6.9 mm (Ø 6.4 mm; n=4); forewing slender; ground colour white, mottled with black; black dots on veins and in terminal area; black patch near base and oblique blackish fascia at about 1/3 to 1/2; fringes white with indistinct dark grey fringe line. Hindwing dark grey, fringes with dark grey base, distal part white.

**Female.** Unknown.

**Male genitalia** (Figs 35–36). Socii long and slender, with apical spine; anterior margin of tegumen with medial process; gnathos broadly tongue-shaped, smooth; valva slender, length 0.61 mm, max width 0.19 mm; densely covered with long hairs in medial part and short setae on ventromedial margin, ventromedial part weakly convex, distal part with ventrally convex and dorsally projected apex, costa strongly sclerotized with indistinct distal dentation; sacculus oval, weakly confined, densely covered with strong setae; saccus short, about 0.24 mm, slender, about same width throughout, apex rounded; phallus 0.95 mm long, slender, uneverted vesica with ca. 0.35 mm long sclerotized apical part, including 3 short cornuti of about 0.22 mm length [hardly discernible in situ].

**Female genitalia.** Unknown.

###### Molecular data.

*Kessleria apenninica* splits into two geographically separated haplogroups, which in our examination – based on limited material – did not reveal any morphological differences. The average intraspecific divergence of the barcode region is considerable with 1.05%, ranging from a minimum of 0% to a maximum of 1.69% (n=4). The minimum distance to the nearest neighbour *Kessleria pyrenaea* is 5.47%.

###### Etymology.

The species name refers to the Apennines where all type specimens have been collected.

###### Distribution.

Only known from the Apennines in Central Italy.

###### Ecology.

Host-plant and early stages unknown, but the species probably feeds on an unidentified broad-leaved *Saxifraga* species growing on steep rocks. The adults have been collected in the last third of July from light. The species occurs in rocky habitat on calcareous soil. Vertical distribution: from about 2100 m to 2200 m.

#### *Kessleria albescens*-group

The *Kessleria albescens*-group is characterized by small and predominantly whitish-coloured species without obvious sexual dichroism or dimorphism (Figs 39–48). The male genitalia are recognizable by the strongly spinous sacculus, the long and stout saccus, and particularly the phallus with two long cornuti with bases of similar length (Figs 49–58). The female genitalia are characterized by the curved entrance of the ductus bursae and the finely granulated sculpture of the entire ductus bursae (Figs 59–66). Larval host-plants, as far as is known, belong to broad-leaved *Saxifraga* spp., e.g. *Saxifraga paniculata* and *Saxifraga incrustata*. Five species are known: *Kessleria albescens*, *Kessleria inexpectata*, *Kessleria helvetica*, *Kessleria klimeschi* and the new species *Kessleria orobiae*.

**Figures 39–44.**
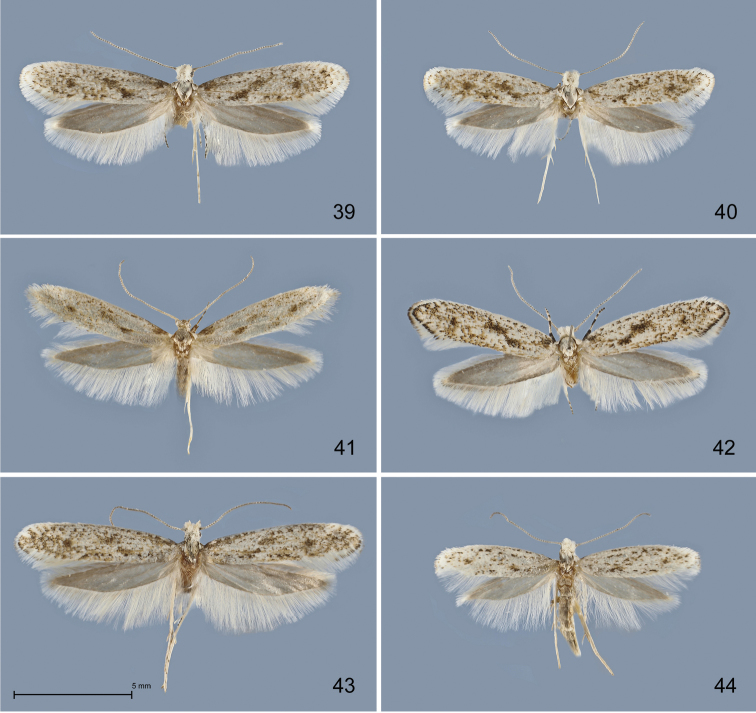
*Kessleria* adults in dorsal view. **39** *Kessleria klimeschi*, ♂, holotype, Italy, Prov. Udine, Montasio, Malga Pecol, 1600 m, 24.6.1989 e.l., leg. Huemer & Tarmann (TLMF) **40** *Kessleria klimeschi*, ♀, paratype, same data, but 3.7.1989 e.l. (TLMF) **41** *Kessleria helvetica*, ♂, holotype, Switzerland, Wallis, Zermatt, 1850 m, 10.8.1980, leg. Whitebread (DNA barcode ID TLMF Lep 01868) (TLMF) **42** *Kessleria helvetica*, ♀, Switzerland, Wallis, Zermatt, Triftschlucht, 1820 m, 10.6.2014 e.l., leg. Schmid (DNA barcode ID TLMF Lep 14996) (TLMF) **43** *Kessleria inexpectata*, ♂, paratype, France, Dep. Alpes Maritimes, Marguareis W-Hang, Navela, 2100–2200 m, 21.–23.7.1990, leg. Huemer & Tarmann (TLMF) **44** *Kessleria inexpectata*, ♀, paratype, same data (TLMF).

**Figures 45–48.**
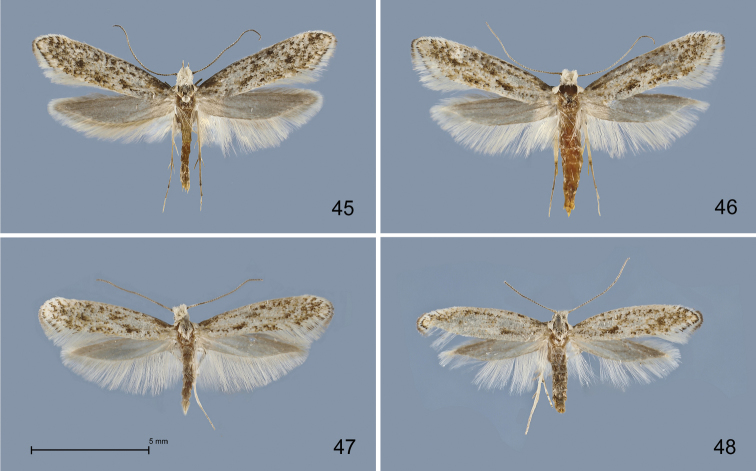
*Kessleria* adults in dorsal view. **45** *Kessleria orobiae* sp. n., ♂, paratype, Zambla Alta – Plassa, 1160 m, 24.6.2013 leg. Mayr (coll. Mayr) **46** *Kessleria orobiae* sp. n., ♀, paratype, same data (coll. Mayr) **47** *Kessleria albescens*, ♂, Italy, Monte Baldo, Bocca di Navene, 14.7.1987, leg. Huemer & Tarmann (DNA barcode ID TLMF Lep 03131) (TLMF) **48** *Kessleria albescens* ♀, same data, but 10.9.1987 e.l. (DNA barcode ID TLMF Lep 01866) (TLMF).

##### Kessleria orobiae sp. n.

Taxon classificationAnimaliaLepidopteraYponomeutidae

http://zoobank.org/86461879-486E-49A4-9004-D35885EAED96

###### Type material.

**Holotype.** ♂, „ITALIA sept. Prov. Bergamo, Alpi Orobie Zambla Alta – Plassa 9°47'48"E, 45°54'12"N 1160 m, 24.6.2013 leg. Huemer“ „DNA Barcode TLMF Lep 09972“ „YPO 160 ♂ P. Huemer“ (TLMF).

**Paratypes. Italy:** 6 ♂, 6 ♀, same data, DNA barcode IDs TLMF Lep 09971, TLMF Lep 09973 (TLMF); 1 ♂, 1 ♀, same data, but e.l. 11.7.2013 (TLMF); 7 ♂, 2 ♀, same data, but leg. Mayr (coll. Mayr, Feldkirch); 1 ♂, Prov. Bergamo, Alpi Orobie, Val d´Arera, 2000 m, 14.–15.8.1992, leg. Huemer (TLMF); 1 ♀, Prov. Bergamo, Alpi Orobie, W. Ca. San Marco, 2100 m, e.l. 31.7.1992, leg. Huemer & Tarmann, DNA barcode ID TLMF Lep 03175 (TLMF).

###### Diagnosis.

**Figures 49–54.**
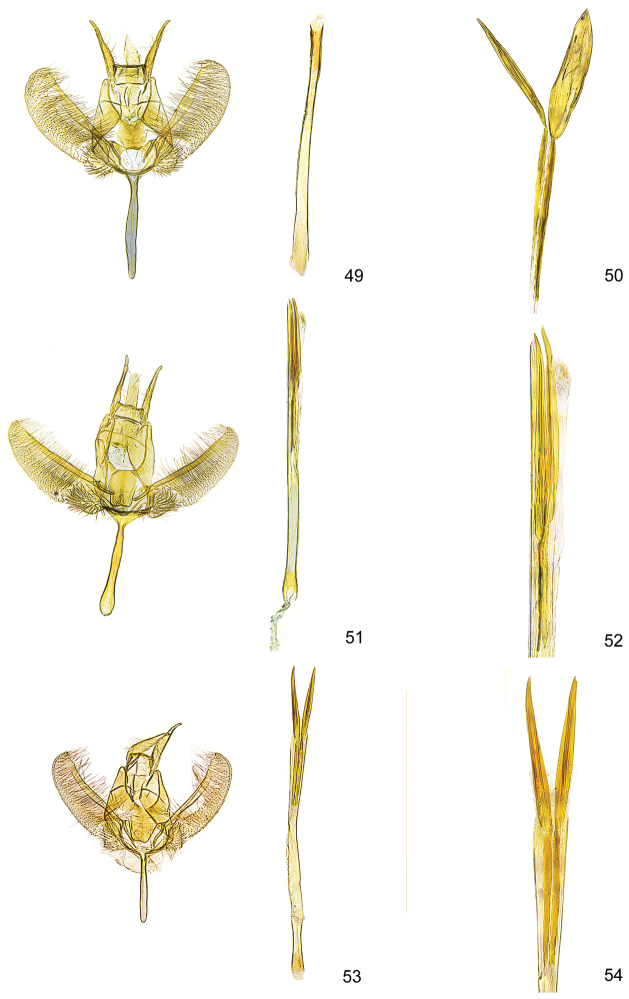
*Kessleria* male genitalia. **49** *Kessleria klimeschi*, holotype, Italy, Prov. Udine, Montasio, Malga Pecol, 1600 m, 24.6.1989 e.l., leg. Huemer & Tarmann, genitalia slide YPO 17 (TLMF) **50** idem, cornuti enlarged **51** *Kessleria helvetica*, holotype, Switzerland, Wallis, Zermatt, 1850 m, 10.8.1980, leg. Whitebread, gen. slide 350 Whitebread (DNA barcode ID TLMF Lep 01868) (TLMF) **52** idem, distal part of phallus enlarged **53** *Kessleria inexpectata*, paratype, France, Dep. Alpes Maritimes, Marguareis W-Hang, Navela, 2100–2200 m, 21.–23.7.1990, leg. Huemer & Tarmann, gen. slide YPO 63 (TLMF) **54** idem, distal part of phallus enlarged.

**Figures 55–58.**
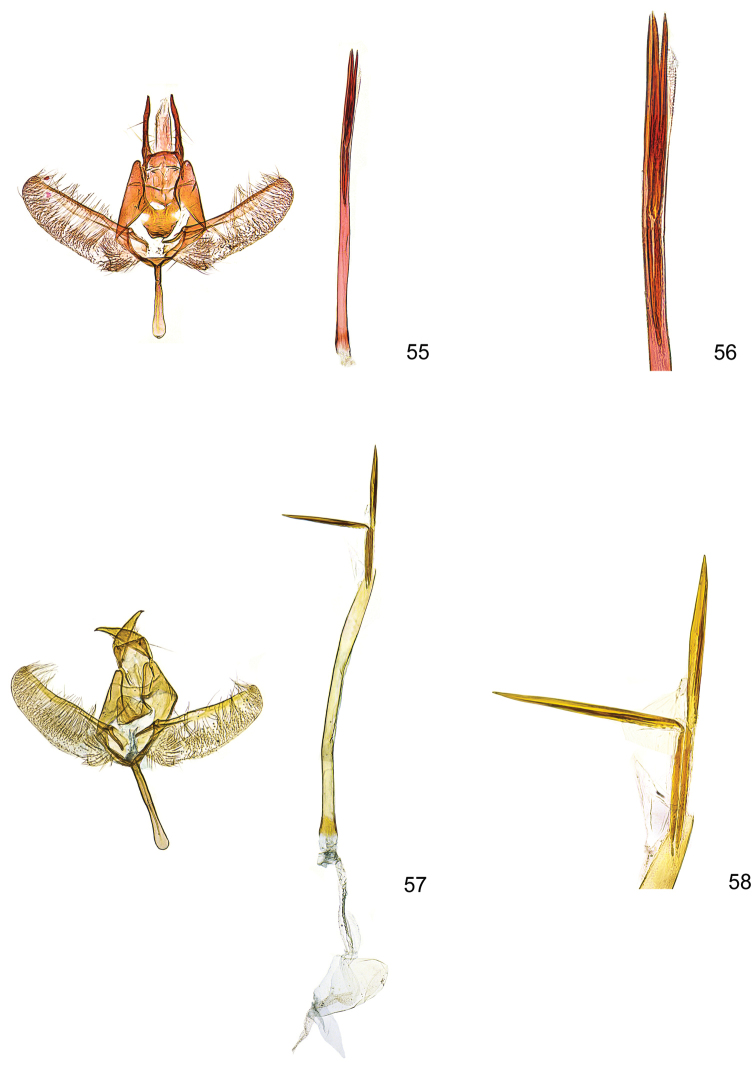
*Kessleria* male genitalia. **55** *Kessleria orobiae* sp. n., holotype, Zambla Alta – Plassa, 1160 m, 24.6.2013 leg. Huemer, gen. slie YPO 160 (TLMF) **56** idem, distal part of phallus enlarged **57** *Kessleria albescens*, ♂, Italy, Monte Baldo, Bocca di Navene, 14.7.1987, leg. Huemer & Tarmann, gen. slide YPO 19 (DNA barcode ID TLMF Lep 03131) (TLMF) **58** idem, distal part of phallus enlarged.

**Figures 59–62.**
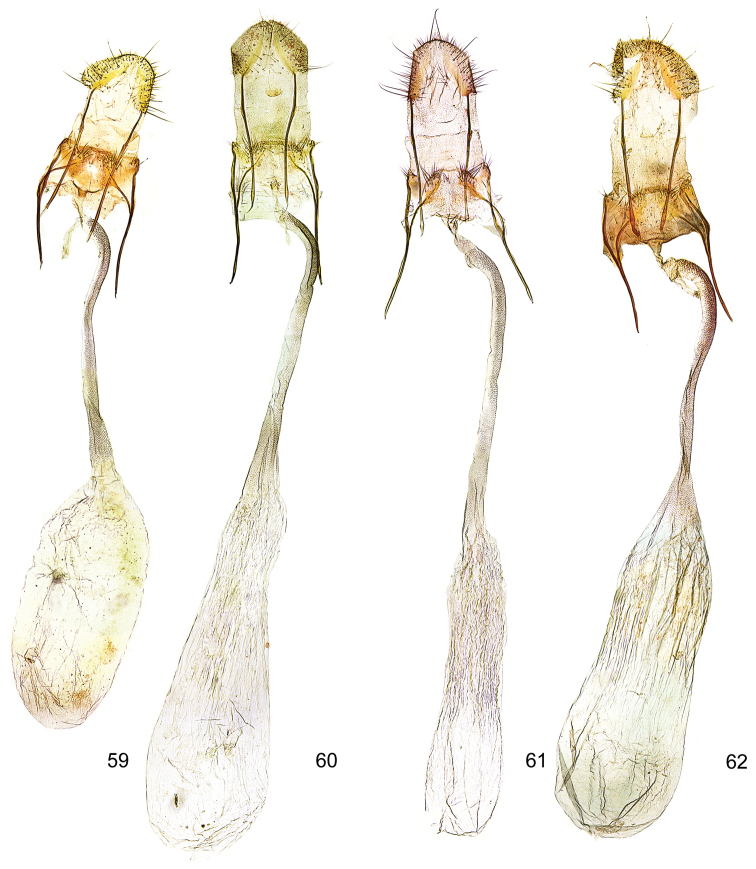
*Kessleria* female genitalia. **59** *Kessleria klimeschi*, paratype, Italy, Prov. Udine, Montasio, Malga Pecol, 1600 m, 24.6.1989 e.l., leg. Huemer & Tarmann, gen. slide YPO 76 (TLMF) **60** *Kessleria inexpectata*, paratype, France, Dep. Alpes Maritimes, Marguareis W-Hang, Navela, 2100–2200 m, 21.–23.7.1990, leg. Huemer & Tarmann, gen. slide YPO 74 (TLMF) **61** *Kessleria orobiae* sp. n., paratype, Zambla Alta – Plassa, 1160 m, 24.6.2013, leg. Huemer, gen. slide YPO 161 (TLMF) **62** *Kessleria albescens*, Italy, Monte Baldo, Bocca di Navene, 14.7.1987, leg. Huemer & Tarmann (TLMF).

**Figures 63–66.**
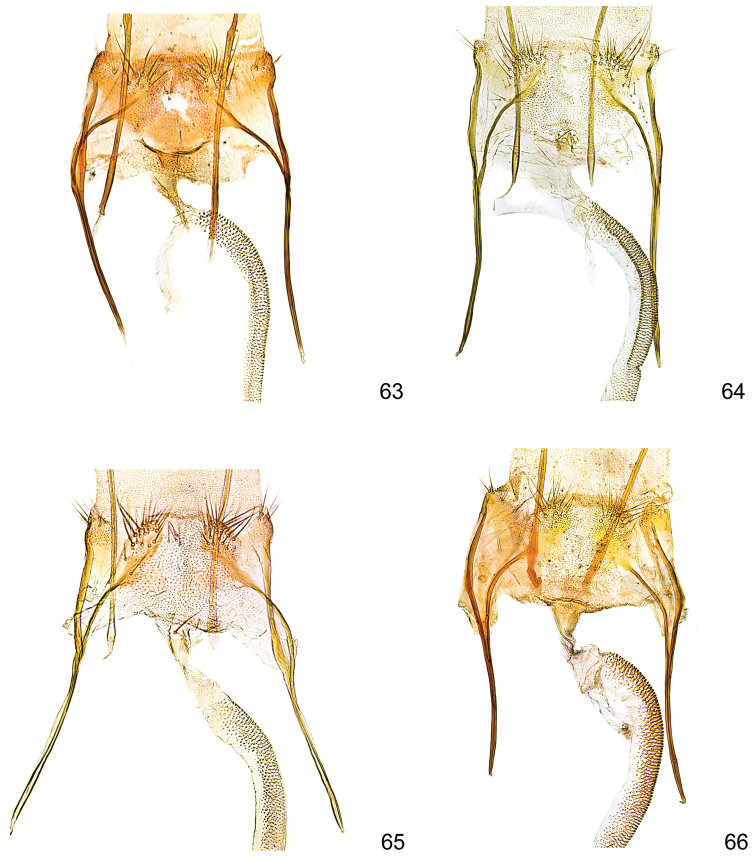
*Kessleria* female genitalia, details of VIII abdominal segment enlarged. **63** *Kessleria klimeschi*, paratype, Italy, Prov. Udine, Montasio, Malga Pecol, 1600 m, 24.6.1989 e.l., leg. Huemer & Tarmann, gen. slide YPO 76 (TLMF) **64** *Kessleria inexpectata*, paratype, France, Dep. Alpes Maritimes, Marguareis W-Hang, Navela, 2100–2200 m, 21.-23.7.1990, leg. Huemer & Tarmann, gen. slide YPO 74 (TLMF) **65** *Kessleria orobiae* sp. n., paratype, Zambla Alta – Plassa, 1160 m, 24.6.2013, leg. Huemer, gen. slide YPO 161 (TLMF) **66** *Kessleria albescens*, Italy, Monte Baldo, Bocca di Navene, 14.7.1987, leg. Huemer & Tarmann (TLMF).

*Kessleria orobiae* largely resembles other taxa of the *Kessleria albescens*-group in wing markings and colour, and cannot be unambiguously separated (Figs 39–48). Similarly, female genitalia exhibit no significant diagnostic characters for discrimination at species level (Figs 59–66, and Huemer and Tarmann 1992). The most reliable diagnostic characters in the species-group are found in the male genitalia (Figs 49–58). *Kessleria orobiae* differs from *Kessleria klimeschi*, *Kessleria albescens* and *Kessleria helvetica* by a much shorter saccus (0.32 mm *vs.* minimum 0.38–0.58 mm) which is only about half the length of the valva compared to the minimum 0.75 times the length of the valva in the other species. In *Kessleria inexpectata* the saccus is slightly longer and furthermore without the apical widening of *Kessleria orobiae*, *Kessleria albescens* and *Kessleria helvetica*. The two needle-shaped cornuti are similar in all species, with the exception of *Kessleria klimeschi* with only one needle-shaped and one sub-ovate cornutus, and *Kessleria helvetica* with cornuti of about 0.50 mm in length. The female genitalia largely resemble other species of the *Kessleria albescens*-group with only quantitative differences, such as a longer ductus bursae than in *Kessleria albescens* (1.6 mm *vs.* 1.3–1.4 mm), and the overall length of genitalia which exceeds *Kessleria klimeschi* (4.2 mm *vs.* 3.5 mm).

###### Description.

**Male** (Fig. 45). Head covered with white hair-like scales; antennae ringed dark grey and whitish; thorax and tegulae mixed dark grey and white, distally predominantly white, particularly tegulae. Forewing length 6.7–7.3 mm (Ø 7.03 mm; n=6); ground colour whitish-grey, intensively mottled with blackish-grey spots all over wing, few ochre-brown scales in dorsal part; blackish-grey patches at base of costa and at end of cell, oblique blackish-grey fascia at about 1/3 to 1/2 narrow and indistinct, medially separated; termen whitish-grey; fringes white, basally with distinct blackish-grey cilia line, apex with small dark grey tip. Hindwing dark grey, fringes with dark grey base, distal part white.

**Female** (Fig. 46). As male. Head covered with white hair-like scales; antennae ringed dark grey and whitish; thorax and tegulae mixed dark grey and white, distally predominantly white, particularly tegulae. Forewing length 5.9–6.6 mm (Ø 8.18 mm; n=5); ground colour whitish-grey, intensively mottled with blackish-grey spots all over wing, few ochre-brown scales in dorsal part; blackish-grey patches at base of costa and at end of cell, oblique blackish-grey fascia at about 1/3 to 1/2 narrow and indistinct, medially separated; termen whitish-grey; fringes white, basally with distinct blackish-grey cilia line, apex with small dark grey tip. Hindwing dark grey, fringes with dark grey base, distal part white.

**Male genitalia** (Figs 55–56). Socii long and slender, with apical spine; anterior margin of tegumen with medial process; gnathos broadly tongue-shaped, smooth; valva slender, length 0.60 mm, maximum width 0.18 mm; densely covered with long hairs in medial part and short setae on ventromedial margin, apical part ventrally convex, costa strongly sclerotized without dentation; sacculus oval, weakly confined, densely covered with strong setae; saccus moderate in length, about 0.32 mm, stout, distally widened with rounded apex; phallus 1.34 mm long, slender, uneverted vesica with ca. 0.70 mm long sclerotized apical part, including two prominent needle-shaped cornuti of about 0.40 mm length.

**Female genitalia** (Figs 61, 65). Genitalia ca. 4.2 mm in length; papilla analis large, densely covered with long setae; apophysis posterior rod like, ca. 0.70 mm, about length of apophysis anterior; apophysis anterior rod like; posterior part bifurcated with straight dorsal and inwardly curved ventral branch; ventral branch descending into patch like sclerite; lamella postvaginalis with sclerotized lateral patches, covered with microtrichia, medially membranous, posterolaterally with hump, covered with some long setae; ostium bursae membranous; antrum weakly developed, ring-like; ductus bursae long, about 1.6 mm, weakly curved in posterior part, from entrance of ductus seminalis to almost transition to corpus bursae densely covered with finely granulous sculpture; sculpture in posterior part nearly bacillary, distally increasingly granulous, entrance to corpus bursae widened, without sculpture; corpus bursae about 1.7 mm in length, saccate, posterior part folded, without signum.

###### Molecular data.

The average intraspecific divergence of the barcode region is low with 0.31%, ranging from a minimum of 0.15% to a maximum of 0.46% (n=5). The minimum distance to the nearest neighbour *Kessleria albescens* is 2.66%, whereas the minimum divergence to *Kessleria inexpectata*, *Kessleria helvetica* and *Kessleria klimeschi* ranges from 3.14% and 3.46% to 9.53%, respectively.

###### Etymology.

The species name refers to the Orobian Alps (Alpi Orobie) in northern Italy, where the type locality is situated.

###### Distribution

**Figure 67.**
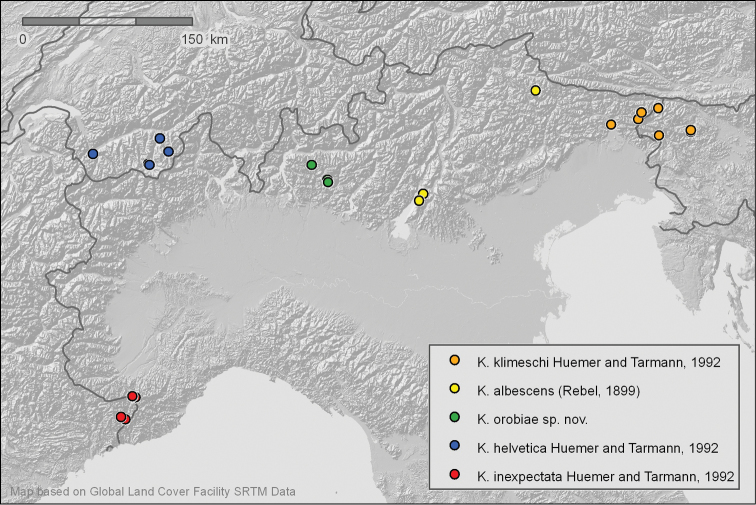
Distribution pattern of the *Kessleria albescens*-group from examined material.

(Fig. 67). Only known from Zambla Alta – Plassa and few nearby localities in the Orobian Alps (Prov. Bergamo, Italy).

###### Ecology.

The larval habits are insufficiently known, but based on our observations, the larva lives in the shoots and as a leaf-miner in basal leaves of *Saxifraga paniculata* and *Saxifraga* sp. Mined leaves are partially spun together and covered with a fine silken web. The adults have been collected from the *Saxifraga*-cushions or nearby rock during the day. In the first few hours of the night they have been observed with a head-lamp flying actively around the larval habitat or sitting near the host-plant. The adult is on the wing from late June to mid-August, depending on altitude and snow coverage. Bred specimens date from mid to late July. *Kessleria orobiae* occurs in rocky habitat both on calcareous and silicous soil. Vertical distribution: from about 1100 m to 2100 m.

#### Established European species of *Kessleria*

A brief overview of established species lists including original description, type locality, type material, references of published images of adults and images of genitalia, and hitherto unpublished molecular data. For extensive generic and species descriptions and diagnoses, see Huemer and Tarmann (1992).

##### Genus *Kessleria* Nowicki, 1864

*Kessleria* Nowicki 1864: 12. Type species: *Kessleria zimmermanni* Nowicki, 1864 by monotypy and original designation.

###### Subgenus *Kessleria* Nowicki, 1864

####### *Kessleria alpicella* (Stainton, 1851)

Tinea (Oecophora!) alpicella [Fischer von Röslerstamm, Mann in litt.] Stainton 1851: 17. Type locality: Europe [?Austria]. Type material: Lectotype (designated by Huemer and Tarmann 1992: 16) (BMNH) [examined].

*Swammerdamia alpicella* Herrich-Schäffer 1855: 272. Type locality: Austria, Niederösterreich, Schneeberg. Type material: Syntypes [not traced]. Homonym and synonym.

Redescription and diagnosis see Huemer and Tarmann 1992: 16–18, figs 1–2 (adult), figs 85–93 (male genitalia), figs 220–221 (female genitalia).

**Molecular data.** *Kessleria alpicella* splits into three geographically separate haplogroups, indicating potential cryptic diversity. The average intraspecific divergence of the barcode region is high with 1.52%, ranging from a minimum of 0% to a maximum of 4.27% (n=12). The minimum distance to the nearest neighbour *Kessleria wehrlii* is 6.9%.

####### *Kessleria mixta* Huemer & Tarmann, 1992

*Kessleria mixta* Huemer and Tarmann 1992: 18. Type locality: Albania, Korab. Type material: Holotype (designated by Huemer and Tarmann 1992: 19) (NHMV) [examined].

Description and diagnosis see Huemer and Tarmann 1992: 18–19, Fig. 3 (adult), figs 94–96 (male genitalia).

**Molecular data.** Unavailable.

**Remarks.** Female unknown.

####### *Kessleria alternans* (Staudinger, 1871)

*Kessleria alternans* Staudinger 1871: 291. Type locality: Switzerland, Graubünden, Sils-Maria. Type material: Lectotype (designated by Friese 1960: 75) (MNHU) [examined].

Redescription and diagnosis see Huemer and Tarmann 1992: 19–22, figs 4–6 (adult), figs 97–102 (male genitalia), figs 223–224 [misidentified figures depicting newly described species in this paper are not listed].

**Molecular data.** The intraspecific divergence of the barcode region is low, ranging from a minimum of 0% to a maximum of 0.32% (mean 0.12%) (n=10). The minimum distance to the nearest neighbour *Kessleria cottiensis* is 2.65%.

**Remarks.** Huemer and Tarmann (1992) already recognized and described a remarkable amount of individual and geographical variation. At that time, this variation was considered as intraspecific, and the authors hesitated to describe further species.

####### *Kessleria wehrlii* Huemer & Tarmann, 1992

*Kessleria wehrlii* Huemer and Tarmann 1992: 22. Type locality: France, Dep. Alpes-Maritimes, Mont Colomb. Type material: Holotype (designated by Huemer and Tarmann 1992: 23) (TLMF) [examined].

Description and diagnosis see Huemer and Tarmann 1992: 22–23, figs 11–12 (adult), figs 109–111 (male genitalia), fig. 211 (8^th^ abdominal segment), fig. 222 (female genitalia).

**Molecular data.** The average intraspecific divergence of the barcode region is 0.0% (n=4). The minimum distance to the nearest neighbour *Kessleria alpmaritimae* is 1.87%.

####### *Kessleria nivescens* Burmann, 1980

*Kessleria nivescens* Burmann 1980: 105. Type locality: Italy, Prov. Verona, Monte Baldo. Type material: Holotype (designated by Burmann 1980: 107) (SMNK) [examined].

Redescription and diagnosis see Huemer and Tarmann 1992: 23–26, figs 13–17 (adult), figs 115–120 (male genitalia), fig. 219 (female genitalia).

**Molecular data.** *Kessleria nivescens* splits into three geographically separate haplogroups. The intraspecific divergence of the barcode region is high, ranging from a minimum of 0% to a maximum of 2.5% (mean 1.09%) (n=14). The minimum distance to the nearest neighbour *Kessleria petrobiella* is 3.29% (mean 4.52%, max. 4.92%).

**Remarks.** Huemer and Tarmann (1992) already recognized and described a considerable amount of individual and geographical variation, particularly in phenotypic appearance, but the authors hesitated to describe further species due to the lack of diagnostic genitalia characters. Molecular data suggest possible cryptic diversity, but further investigations are required.

####### *Kessleria petrobiella* (Zeller, 1868)

*Scythropia petrobiella* Zeller 1868: 607. Type locality: Slovenia, [Log Pod Mangrtom]. Type material: Lectotype (designated by Huemer and Tarmann 1992: 37) (BMNH) [examined].

Redescription and diagnosis see Huemer and Tarmann 1992: 37–39, figs 32–33 (adult), figs 156–161 (male genitalia), fig. 229 (female genitalia).

**Molecular data.** The intraspecific divergence of the barcode region is 0% (n=4). The minimum distance to the nearest neighbour *Kessleria nivescens* is 3.29%.

####### *Kessleria macedonica* Huemer & Tarmann, 1992

*Kessleria macedonica* Huemer and Tarmann 1992: 26. Type locality: Kosovo/Macedonia, Shar Planina, Crni vrh. Type material: Holotype (designated by Huemer and Tarmann 1992: 26) (ZSM) [examined].

Description and diagnosis see Huemer and Tarmann 1992: 26–27, fig. 18 (adult), figs 121–123 (male genitalia).

**Molecular data.** Unavailable.

**Remarks.** Female unknown.

####### *Kessleria albanica* Friese, 1960

*Kessleria albanica* Friese 1960: 68. Type locality: Albania, Nikai. Type material: Holotype (designated by Friese 1960: 68) (ZSM) [examined].

Redescription and diagnosis see Huemer and Tarmann 1992: 27–28, fig. 19 (adult), figs 124–125 (male genitalia).

**Molecular data.** *Kessleria albanica* splits into three geographically separate haplogroups, indicating potential cryptic diversity. The average intraspecific divergence of the barcode region is high with 2.08%, ranging from a minimum of 0% to a maximum of 3.12% (n=5). The minimum distance to the nearest neighbour *Kessleria burmanni* is 9.29%.

**Remarks.** Female unknown.

####### *Kessleria burmanni* Huemer & Tarmann, 1992

*Kessleria burmanni* Huemer and Tarmann 1992: 28. Type locality: Austria, Nordtirol, Nordkette. Type material: Holotype (designated by Huemer and Tarmann 1992: 30) (TLMF) [examined].

Description and diagnosis see Huemer and Tarmann 1992: 28–30, figs 20–22 (adult), figs 126–131 (male genitalia), fig. 225 (female genitalia), figs 63–65 (SEM egg structures).

**Molecular data.** The average intraspecific divergence of the barcode region is 0.0% (n=6). The minimum distance to the nearest neighbour *Kessleria hauderi* is 7.61%.

####### *Kessleria insubrica* Huemer & Tarmann, 1993

*Kessleria insubrica* Huemer and Tarmann 1993: 41. Type locality: Italy, Prov. Bergamo, Val d´Arera. Type material: Holotype (designated by Huemer and Tarmann 1993: 45) (TLMF) [examined].

Description and diagnosis see Huemer and Tarmann 1993: 41–46, fig. 1 (adult), figs 5–6 (female genitalia).

**Molecular data.** The average intraspecific divergence of the barcode region is low with 0.08%, ranging from a minimum of 0% to a maximum of 0.15% (n=4). The minimum distance to the nearest neighbour *Kessleria burmanni* is 8.95%.

**Remarks.** Male unknown.

####### *Kessleria hauderi* Huemer & Tarmann, 1992

*Kessleria hauderi* Huemer and Tarmann 1992: 31. Type locality: Austria, Steiermark, Eisenerzer Reichenstein. Type material: Holotype (designated by Huemer and Tarmann 1992: 32) (TLMF) [examined].

Description and diagnosis see Huemer and Tarmann 1992: 21–33, figs 23–24 (adult), figs 132–137 (male genitalia), fig. 227 (female genitalia).

**Molecular data.** The average intraspecific divergence of the barcode region is 0% (n=2). The minimum distance to the nearest neighbour *Kessleria burmanni* is 7.61%.

####### *Kessleria diabolica* Huemer & Tarmann, 1992

*Kessleria diabolica* Huemer and Tarmann 1992: 33. Type locality: Spain, Prov. Avila, Sierra de Gredos. Type material: Holotype (designated by Huemer and Tarmann 1992: 33) (ZMUC) [examined].

Description and diagnosis see Huemer and Tarmann 1992: 33, fig. 25 (adult), figs 138–140 (male genitalia).

**Molecular data.** Unavailable.

**Remarks.** Female unknown.

####### *Kessleria brevicornuta* Huemer & Tarmann, 1992

*Kessleria brevicornuta* Huemer and Tarmann 1992: 34. Type locality: Spain, Prov. Avila, Sierra de Gredos. Type material: Holotype (designated by Huemer and Tarmann 1992: 34) (coll. Arenberger, Vienna) [examined].

Description and diagnosis see Huemer and Tarmann 1992: 34–35, figs 26–27 (adult), figs 141–143 (male genitalia), fig. 235 (female genitalia).

**Molecular data.** Unavailable.

####### *Kessleria pyrenaea* Friese, 1960

*Kessleria pyrenaea* Friese 1960: 76. Type locality: France, Dép. Pyrénées-Orientales, Mt. Canigou. Type material: Holotype (designated by Friese 1960: 76) (NHMV) [examined].

Redescription and diagnosis see Huemer and Tarmann 1992: 35, fig. 28 (adult), figs 144–146 (male genitalia).

**Molecular data.** The intraspecific divergence of the barcode region is unknown (n=1). The minimum distance to the nearest neighbour *Kessleria apenninica* is 5.47%.

**Remarks.** Female unknown. The identity of the sequenced specimen is doubtful.

####### *Kessleria brachypterella* Huemer & Tarmann, 1992

*Kessleria brachypterella* Huemer and Tarmann 1992: 36. Type locality: France, Dép. Hautes-Pyrénées, Pic du Midi de Bigorre. Type material: Holotype (designated by Huemer and Tarmann 1992: 34) (BMNH) [examined].

Description and diagnosis see Huemer and Tarmann 1992: 36–37, figs 29–30 (adult), figs 147–149 (male genitalia), fig. 228 (female genitalia).

**Molecular data.** Unavailable.

####### *Kessleria zimmermanni* Nowicki, 1864

*Kessleria zimmermanni* Nowicki 1864: 13. Type locality: Poland, Tatra mts., ?Kopa Magury. Type material: Lectotype (designated by Huemer and Tarmann 1992: 39) (SDEI) [examined].

*Kessleria tatrica* Friese 1960: 71. Type locality: [?Poland], Tatra mts. Type material: Holotype (designated by Friese 1960: 71) (NHMV).

Redescription and diagnosis see Huemer and Tarmann 1992: 39–42, figs 32–33 (adult), figs 162–167 (male genitalia), fig. 217 (8^th^ abdominal segment), fig. 226 (female genitalia).

**Molecular data.** The intraspecific divergence of the barcode region is 0% (n=3). The minimum distance to the nearest neighbour *Kessleria petrobiella* is 5.73%.

####### *Kessleria albomaculata* Huemer & Tarmann, 1992

*Kessleria albomaculata* Huemer and Tarmann 1992: 42. Type locality: France, Dép. Hautes-Pyrénées, Cauterets. Type material: Holotype (designated by Huemer and Tarmann 1992: 42) (MNCN) [examined].

Description and diagnosis see Huemer and Tarmann 1992: 42–43, fig. 36 (adult), figs 153–155 (male genitalia), fig. 216 (8^th^ abdominal segment).

**Molecular data.** The intraspecific divergence of the barcode region is unknown (n=1). The minimum distance to the nearest neighbour *Kessleria petrobiella* is 6.76%.

**Remarks.** Female unknown.

####### *Kessleria caflischiella* (Frey, 1880)

*Swammerdamia caflischiella* Frey 1880: 344. Type locality: Switzerland, Wallis, ?Gamsen. Type material: Holotype (designated by Frey 1880: 344) (BMNH) [examined].

Redescription and diagnosis see Huemer and Tarmann 1992: 49–51, figs 45–46 (adult), figs 191–196 (male genitalia), fig. 229 (female genitalia).

**Molecular data.** The average intraspecific divergence of the barcode region is low with 0.04%, ranging from a minimum of 0% to a maximum of 0.15% (n=8). The minimum distance to the nearest neighbour *Kessleria alpmaritimae* is 6.39%.

####### *Kessleria klimeschi* Huemer & Tarmann, 1992

*Kessleria klimeschi* Huemer and Tarmann 1992: 47. Type locality: Italy, Prov. Udine. Montasio, Malga Pecol. Type material: Holotype (designated by Huemer and Tarmann 1992: 48) (TLMF) [examined].

Description and diagnosis see Huemer and Tarmann 1992: 47–49, figs 43–44 (adult), figs 185–190 (male genitalia), fig. 218 (8^th^ abdominal segment), fig. 233 (female genitalia).

**Molecular data.** The average intraspecific divergence of the barcode region is low with 0.06%, ranging from a minimum of 0% to a maximum of 0.15% (n=5). The minimum distance to the nearest neighbour *Kessleria inexpectata* is 8.83%.

####### *Kessleria helvetica* Huemer & Tarmann, 1992

*Kessleria helvetica* Huemer and Tarmann 1992: 46. Type locality: Switzerland, Wallis, Zermatt. Type material: Holotype (designated by Huemer and Tarmann 1992: 47) (TLMF) [examined].

Description and diagnosis see Huemer and Tarmann 1992: 46–47, figs 41–42 (adult), Figs 179–184 (male genitalia), fig. 232 (female genitalia).

**Molecular data.** The average intraspecific divergence of the barcode region is unknown (n=1). *Kessleria helvetica* overlaps in the barcode with a haplogroup of topotypical *Kessleria inexpectata*, but diagnostic morphological characters indicate species status. The minimum distance to a further haplogroup of *Kessleria inexpectata* is 1.77%.

####### *Kessleria inexpectata* Huemer & Tarmann, 1992

*Kessleria inexpectata* Huemer and Tarmann 1992: 45. Type locality: France, Dep. Alpes Maritimes, Marguareis. Type material: Holotype (designated by Huemer and Tarmann 1992: 46) (TLMF) [examined].

Description and diagnosis see Huemer and Tarmann 1992: 45–46, figs 39–40 (adult), figs 173–178 (male genitalia), fig. 231 (female genitalia).

**Molecular data.** *Kessleria inexpectata* splits into two major haplogroups. The average intraspecific divergence of the barcode region within the haplogroup of topotypical specimens is low with 0.16%, ranging from a minimum of 0% to a maximum of 0.32% (n=4) whereas the average intraspecific variation within the second haplogroup is considerable with 0.84% (maximum 1.68%). The mean intraspecific divergence of the entire sample is 1.42% (maximum 2.18%). The haplogroup of the topotypical population overlaps with *Kessleria helvetica*. The minimum distance to *Kessleria orobiae* is 3.14%.

####### *Kessleria albescens* (Rebel, 1899)

*Hofmannia albescens* Rebel 1899: 177. Type locality: Italy, South Tyrol, Bozen. Type material: Lectotype (designated by Friese 1960: 72) (NHMV) [examined].

Description and diagnosis see Huemer and Tarmann 1992: 43–44, figs 37–38 (adult), figs 168–172 (male genitalia), fig. 230 (female genitalia).

**Molecular data.** The average intraspecific divergence of the barcode region is 0% (n=3). The minimum distance to the nearest neighbour *Kessleria orobiae* is 2.66%.

###### Subgenus *Hofmannia* Heinemann & Wocke, 1877

####### *Kessleria saxifragae* (Stainton, 1868)

*Zelleria saxifragae* Stainton 1868: 139. Type locality: Austria, Nordtirol, Kaisergebirge. Type material: ?Syntypes (examined by Huemer and Tarmann 1992: 53) (BMNH) [examined].

Description and diagnosis see Huemer and Tarmann 1992: 51–54, figs 47–50 (adult), figs 197–202 (male genitalia), fig. 236 (female genitalia).

**Molecular data.** The average intraspecific divergence of the barcode region is low with 0.43%, ranging from a minimum of 0% to a considerable maximum of 1.28% (n=20). The minimum distance to the nearest neighbour *Zelleria celastrusella* Kearfott, 1903, from North America is 6.22%, and the minimum distance to the congeneric *Kessleria fasciapennella* is 7.21%.

####### *Kessleria fasciapennella* (Stainton, 1849)

*Zelleria fasciapennella* Stainton 1849: 80. Type locality: GB, Scotland, Edinburgh, Pentland hills. Type material: Lectotype (designated by Huemer and Tarmann 1992: 54) (BMNH) [examined].

*Kessleria longipenella* Friese 1960: 83. Type locality: Russia, Karelia, S Petrosawodsk. Type material. Holotype (designated by Friese 1960: 83) (SDEI).

Description and diagnosis see Huemer and Tarmann 1992: 54–56, figs 52–54 (adult), figs 203–208 (male genitalia), fig. 237 (female genitalia).

**Molecular data.** The average intraspecific divergence of the barcode region is low with 0.04%, ranging from a minimum of 0% to a maximum of 0.15% (n=8). The minimum distance to the nearest neighbour *Zelleria celastrusella* Kearfott, 1903, from North America is 6.58%, and the minimum distance to the congeneric *Kessleria saxifragae* is 7.21%.

## Discussion

Our study proves the advantage of an integrative taxonomic approach, initially based on morphology, with molecular data supplemented as an additional tool for delimitation of cryptic species. Even within genera of European Lepidoptera which had been considered as well explored, cryptic diversity seems much more widespread than hitherto estimated. Recent molecular studies have proven the existence of a remarkable amount of cryptic species in several genera or species-groups, e.g. *Callisto* (Kirichenko et al. 2015), *Stigmella* (Nieukerken et al. 2012), *Olethreutes* (Segerer et al. 2010), *Elachista* (Mutanen et al. 2013), *Eulamprotes* (Huemer et al. 2013), *Sattleria* (Huemer and Hebert 2011, Huemer and Timossi 2014), *Caryocolum* (Huemer et al. 2014) and *Coleophora* (Baldizzone et al. 2014, Tabell and Baldizzone 2014). Similarly, the proportion of unnamed species in *Kessleria* is high, adding about 20% to the hitherto described species diversity, not including several further yet unresolved possible candidates of cryptic diversity. We expect to find such additional overlooked taxa in e.g. *Kessleria alpicella* and *Kessleria albanica*, but additional material is needed to resolve this. Outside Europe, the species diversity of *Kessleria* cannot even be estimated at the present time, with the Chinese *Kessleria nivosa* (Meyrick, 1938) as the only known congeneric species from Asia, and *Kessleria parnassiae* (Braun, 1940), a close relative of *Kessleria fasciapennella*, from North America. Particularly the Asian fauna of *Kessleria* may prove diverse, e.g. indicative of which is an extraordinary diversity of potential *Kessleria* host-plants in China with 216 out of about 450 worldwide known species of *Saxifraga* (139 endemic) and 63 out of 70 *Parnassia* spp. (49 endemic) (Jintang et al. 2001).

Most of the newly described species belong to complexes of closely related species with strictly allopatric distribution patterns. *Kessleria cottiensis*, *Kessleria dimorpha* and *Kessleria alpmaritimae* are morphologically and genetically most similar to *Kessleria alternans* and *Kessleria wehrlii*, forming a separate species group in *Kessleria* (Fig. 32). Similarly, *Kessleria orobiae* along with *Kessleria albescens*, *Kessleria klimeschi*, *Kessleria helvetica* and *Kessleria inexpectata* belong to a species-group of cryptic allopatric taxa (Fig. 67). All these taxa are extremely similar in external and internal morphology. This is a phenomenon well known from the related genus *Yponomeuta*, namely the *Yponomeuta cagnagellus* species-complex which includes morphologically virtually indistinguishable species (Bakker et al. 2008), which furthermore often share DNA barcodes. Evolution and reproductive isolation in this genus was likely driven by specific host-plant associations and sex pheromones (Menken 1981, Menken et al. 1992, Menken 1996, Löfstedt 1991, Turner et al. 2010). Unlike *Yponomeuta*, barcode sharing seems to be a rare exception in *Kessleria*, only observed in *Kessleria helvetica* and *Kessleria inexpectata* so far, and indicating possible introgression or recent speciation. DNA barcode divergence to the nearest neighbour is considerable in *Kessleria* with roughly 2–3% distance in sister species, rising to about 6–9% between morphologically well separated taxa (Table 1, Fig. 1). If reflected by at least one supplementing morphological character stage we consider barcode divergence of roughly 2% as taxonomically relevant. These taxa are described as cryptic species and not subspecies, although such decisions are prone to subjectivity (Hausmann and Huemer 2011, Huemer and Mayr in press, Mutanen et al. 2012).

A similar extent of interspecific divergence in allopatric sister species is also known from other Lepidoptera with geographically restricted alpine distribution patterns, e.g. *Sattleria* (Huemer and Hebert 2011) and *Sciadia* (Huemer and Hausmann 2009). The timing of radiation of these and *Kessleria* is unknown, but estimations of substitution rates of COI indicate that divergences of 1.0–2.5% correspond to divergence times of roughly one million years (Kandul et al. 2004, Hausmann et al. 2011). Even though such estimations should be considered with caution, they indicate that several well separated species of *Kessleria* may have diverged already in the lower Pliocene (5.3 mya–1.8 mya) while others, such as four out of five newly described species, are possibly of younger origin. Speciation in these taxa was likely reinforced by climatic oscillations in the late Pliocene and during the Pleistocene, with unglaciated but highly isolated refugial areas, particularly in the southern Alps, an area well documented as a hotspot for endemic Lepidoptera (Huemer 1998). The widespread female flightlessness may have been crucial for reducing dispersal and interrupting gene flow, particularly in maternally inherited mitochondrial DNA, thereby expediting the speciation processes. Host-plant relationship itself seems of limited importance for speciation processes in *Kessleria* as host specificity is moderately pronounced and host-plants are regularly much more widespread than their consumers. E.g. *Saxifraga paniculata* as one of the major host-plants of species of the *Kessleria albescens*-group is widely distributed in the Alps and other European mountain systems, but the *Kessleria* spp. are allopatrically distributed in a small section of the southern Alps. Summing up, further in-depth phylogenetic studies will be necessary to finally identify drivers of speciation in *Kessleria*.

## Supplementary Material

XML Treatment for Kessleria cottiensis

XML Treatment for Kessleria dimorpha

XML Treatment for Kessleria alpmaritimae

XML Treatment for Kessleria apenninica

XML Treatment for Kessleria orobiae

## Supplementary materials

Supplementary material 1 Sample information for specimens used in this study. Data type: Species data Explanation note: Details of collecting data, images, sequences, and trace files for the barcoded specimens are available in the public BOLD dataset “DS-LEAKE”, accessed at http://dx.doi.org/10.5883/DS-LEAKE This dataset is made available under the Open Database License (http://opendatacommons.org/licenses/odbl/1.0/). The Open Database License (ODbL) is a license agreement intended to allow users to freely share, modify, and use this Dataset while maintaining this same freedom for others, provided that the original source and author(s) are credited.Peter Huemer, Marko Mutanen
