# Supplementary material for: Alpha taxonomy of the genus Kessleria Nowicki, 1864, revisited in light of DNA-barcoding (Lepidoptera, Yponomeutidae)
Source: Zookeys. 2015 May 11;(503):89–133. doi: 10.3897/zookeys.503.9590 (PMC4440273; doi:10.3897/zookeys.503.9590)
Supplement: Supplementary material 1 — Sample information for specimens used in this study. [file zookeys-503-089-s001.doc]

**Supplement 1:** List of species names, sample-IDs, process-IDs, BINs, COI-5P sequence length and trace counts (from BOLD database).

| **Species** | **Sample ID** | **Process ID** | **BIN** | **COI-5P Seq. Length** | **COI-5P Trace Count** |
| --- | --- | --- | --- | --- | --- |
| Kessleria albanica | TLMF Lep 08925 | PHLAI430-13 | BOLD:ACC5096 | 658[0n] | 2 |
| Kessleria albanica | TLMF Lep 08670 | PHLAH866-12 | BOLD:ACC5095 | 658[0n] | 2 |
| Kessleria albanica | TLMF Lep 08934 | PHLAI439-13 | BOLD:ACC5095 | 567[0n] | 2 |
| Kessleria albanica | TLMF Lep 08668 | PHLAH864-12 | BOLD:ACE5626 | 658[0n] | 2 |
| Kessleria albanica | TLMF Lep 08669 | PHLAH865-12 | BOLD:ACC5096 | 658[0n] | 2 |
| Kessleria albescens | TLMF Lep 01867 | PHLAB1067-10 | BOLD:AAQ0978 | 658[0n] | 4 |
| Kessleria albescens | TLMF Lep 01866 | PHLAB1066-10 | BOLD:AAQ0978 | 658[0n] | 6 |
| Kessleria albescens | TLMF Lep 03131 | PHLAD146-11 | BOLD:AAQ0978 | 641[0n] | 4 |
| Kessleria albomaculata | TLMF Lep 08303 | PHLAH484-12 | BOLD:ACA9511 | 658[0n] | 2 |
| Kessleria alpicella | TLMF Lep 08671 | PHLAH867-12 | BOLD:ACC5231 | 658[0n] | 2 |
| Kessleria alpicella | TLMF Lep 08923 | PHLAI428-13 | BOLD:AAU2222 | 658[0n] | 2 |
| Kessleria alpicella | TLMF Lep 08935 | PHLAI440-13 | BOLD:AAU2222 | 658[0n] | 2 |
| Kessleria alpicella | TLMF Lep 03509 | PHLAD334-11 | BOLD:AAU2222 | 658[0n] | 2 |
| Kessleria alpicella | TLMF Lep 11079 | LEATB902-13 | BOLD:ACE8812 | 658[0n] | 2 |
| Kessleria alpicella | TLMF Lep 03145 | PHLAD160-11 | BOLD:ACE8812 | 658[0n] | 4 |
| Kessleria alpicella | TLMF Lep 03147 | PHLAD162-11 | BOLD:ACE8812 | 658[0n] | 4 |
| Kessleria alpicella | TLMF Lep 03507 | PHLAD332-11 | BOLD:AAU2222 | 658[0n] | 2 |
| Kessleria alpicella | TLMF Lep 08926 | PHLAI431-13 | BOLD:AAU2222 | 658[0n] | 2 |
| Kessleria alpicella | TLMF Lep 03146 | PHLAD161-11 | BOLD:ACE8812 | 658[0n] | 4 |
| Kessleria alpicella | TLMF Lep 12213 | LEATE801-13 | BOLD:ACE8812 | 658[0n] | 2 |
| Kessleria alpicella | TLMF Lep 12214 | LEATE802-13 | BOLD:ACE8812 | 658[0n] | 2 |
| Kessleria alpmaritimae | TLMF Lep 01850 | PHLAB1050-10 | BOLD:AAP0591 | 658[0n] | 2 |
| Kessleria alpmaritimae | TLMF Lep 01851 | PHLAB1051-10 | BOLD:AAP0591 | 636[0n] | 4 |
| Kessleria alpmaritimae | TLMF Lep 03100 | PHLAD115-11 | BOLD:AAP0591 | 658[0n] | 4 |
| Kessleria alpmaritimae | TLMF Lep 03102 | PHLAD117-11 | BOLD:AAP0591 | 658[0n] | 4 |
| Kessleria alpmaritimae | TLMF Lep 03103 | PHLAD118-11 | BOLD:AAP0591 | 658[0n] | 4 |
| Kessleria alpmaritimae | TLMF Lep 03101 | PHLAD116-11 | BOLD:AAP0591 | 658[0n] | 4 |
| Kessleria alternans | TLMF Lep 15472 | LEATH260-14 | BOLD:AAP9518 | 658[0n] | 2 |
| Kessleria alternans | TLMF Lep 04066 | PHLAD701-11 | BOLD:AAP9518 | 658[0n] | 2 |
| Kessleria alternans | TLMF Lep 01849 | PHLAB1049-10 | BOLD:AAP9518 | 658[0n] | 4 |
| Kessleria alternans | TLMF Lep 15474 | LEATH262-14 | BOLD:AAP9518 | 658[0n] | 2 |
| Kessleria alternans | TLMF Lep 03098 | PHLAD113-11 | BOLD:AAP9518 | 658[0n] | 4 |
| Kessleria alternans | TLMF Lep 03099 | PHLAD114-11 | BOLD:AAP9518 | 658[0n] | 4 |
| Kessleria alternans | TLMF Lep 01847 | PHLAB1047-10 | BOLD:AAP9518 | 658[0n] | 4 |
| Kessleria alternans | TLMF Lep 04067 | PHLAD702-11 | BOLD:AAP9518 | 658[0n] | 2 |
| Kessleria alternans | TLMF Lep 01848 | PHLAB1048-10 | BOLD:AAP9518 | 658[0n] | 4 |
| Kessleria alternans | TLMF Lep 01846 | PHLAB1046-10 | BOLD:AAP9518 | 658[0n] | 4 |
| Kessleria apenninica | TLMF Lep 01663 | PHLAB863-10 | BOLD:AAO3363 | 658[0n] | 2 |
| Kessleria apenninica | TLMF Lep 01664 | PHLAB864-10 | BOLD:AAO3363 | 658[0n] | 2 |
| Kessleria apenninica | TLMF Lep 01661 | PHLAB861-10 | BOLD:AAO3363 | 655[0n] | 2 |
| Kessleria apenninica | TLMF Lep 01662 | PHLAB862-10 | BOLD:AAO3363 | 655[0n] | 2 |
| Kessleria burmanni | TLMF Lep 03121 | PHLAD136-11 | BOLD:AAQ0979 | 658[0n] | 4 |
| Kessleria burmanni | TLMF Lep 01859 | PHLAB1059-10 | BOLD:AAQ0979 | 658[0n] | 4 |
| Kessleria burmanni | TLMF Lep 03118 | PHLAD133-11 | BOLD:AAQ0979 | 658[0n] | 3 |
| Kessleria burmanni | TLMF Lep 03119 | PHLAD134-11 | BOLD:AAQ0979 | 658[0n] | 4 |
| Kessleria burmanni | TLMF Lep 01860 | PHLAB1060-10 | BOLD:AAQ0979 | 658[0n] | 4 |
| Kessleria burmanni | TLMF Lep 03120 | PHLAD135-11 | BOLD:AAQ0979 | 658[0n] | 4 |
| Kessleria caflischiella | TLMF Lep 03132 | PHLAD147-11 | BOLD:AAK3930 | 658[0n] | 4 |
| Kessleria caflischiella | TLMF Lep 04064 | PHLAD699-11 | BOLD:AAK3930 | 658[0n] | 2 |
| Kessleria caflischiella | TLMF Lep 04065 | PHLAD700-11 | BOLD:AAK3930 | 658[0n] | 2 |
| Kessleria caflischiella | TLMF Lep 01869 | PHLAB1069-10 | BOLD:AAK3930 | 658[0n] | 4 |
| Kessleria caflischiella | TLMF Lep 11619 | LEATE207-13 | BOLD:AAK3930 | 658[0n] | 2 |
| Kessleria caflischiella | TLMF Lep 03133 | PHLAD148-11 | BOLD:AAK3930 | 658[0n] | 4 |
| Kessleria caflischiella | TLMF Lep 01870 | PHLAB1070-10 | BOLD:AAK3930 | 658[0n] | 4 |
| Kessleria caflischiella | TLMF Lep 00790 | PHLAA750-09 | BOLD:AAK3930 | 658[0n] | 2 |
| Kessleria cottiensis | TLMF Lep 03107 | PHLAD122-11 | BOLD:ABZ2213 | 658[0n] | 4 |
| Kessleria cottiensis | TLMF Lep 03106 | PHLAD121-11 | BOLD:ABZ2213 | 658[0n] | 4 |
| Kessleria cottiensis | TLMF Lep 03108 | PHLAD123-11 | BOLD:ABZ2213 | 658[0n] | 4 |
| Kessleria cottiensis | TLMF Lep 03142 | PHLAD157-11 | BOLD:ABZ2213 | 658[0n] | 4 |
| Kessleria cottiensis | TLMF Lep 03144 | PHLAD159-11 | BOLD:ABZ2213 | 658[0n] | 4 |
| Kessleria dimorpha | TLMF Lep 01759 | PHLAB959-10 | BOLD:AAO3364 | 658[0n] | 2 |
| Kessleria dimorpha | TLMF Lep 01756 | PHLAB956-10 | BOLD:AAO3364 | 658[0n] | 2 |
| Kessleria dimorpha | TLMF Lep 01757 | PHLAB957-10 | BOLD:AAO3364 | 658[0n] | 2 |
| Kessleria dimorpha | TLMF Lep 01758 | PHLAB958-10 | BOLD:AAO3364 | 658[0n] | 2 |
| Kessleria fasciapennella | MM00490 | LEFIB126-10 | BOLD:AAF3317 | 658[0n] | 2 |
| Kessleria fasciapennella | TLMF Lep 03134 | PHLAD149-11 | BOLD:AAF3317 | 658[0n] | 4 |
| Kessleria fasciapennella | MM00865 | LEFIB327-10 | BOLD:AAF3317 | 658[0n] | 2 |
| Kessleria fasciapennella | TLMF Lep 01871 | PHLAB1071-10 | BOLD:AAF3317 | 658[0n] | 4 |
| Kessleria fasciapennella | TLMF Lep 01874 | PHLAB1074-10 | BOLD:AAF3317 | 658[0n] | 4 |
| Kessleria fasciapennella | MM06404 | LEFID464-10 | BOLD:AAF3317 | 658[0n] | 2 |
| Kessleria fasciapennella | TLMF Lep 01872 | PHLAB1072-10 | BOLD:AAF3317 | 658[0n] | 4 |
| Kessleria fasciapennella | TLMF Lep 01873 | PHLAB1073-10 | BOLD:AAF3317 | 658[0n] | 4 |
| Kessleria hauderi | TLMF Lep 03125 | PHLAD140-11 | BOLD:AAV6686 | 658[0n] | 4 |
| Kessleria hauderi | TLMF Lep 03124 | PHLAD139-11 | BOLD:AAV6686 | 658[0n] | 4 |
| Kessleria helvetica | TLMF Lep 14996 | LASTS544-14 | BOLD:ABZ6230 | 658[0n] | 2 |
| Kessleria inexpectata | TLMF Lep 01920 | PHLAB1120-10 | BOLD:AAO9389 | 658[0n] | 2 |
| Kessleria inexpectata | TLMF Lep 03127 | PHLAD142-11 |  | 307[0n] | 2 |
| Kessleria inexpectata | TLMF Lep 01864 | PHLAB1064-10 | BOLD:ABZ6230 | 658[0n] | 4 |
| Kessleria inexpectata | TLMF Lep 01921 | PHLAB1121-10 | BOLD:AAO9389 | 658[0n] | 2 |
| Kessleria inexpectata | TLMF Lep 03139 | PHLAD154-11 | BOLD:AAO9389 | 658[0n] | 4 |
| Kessleria inexpectata | TLMF Lep 02904 | PHLAC869-10 | BOLD:AAO9389 | 658[0n] | 2 |
| Kessleria inexpectata | TLMF Lep 01865 | PHLAB1065-10 | BOLD:ABZ6230 | 658[0n] | 4 |
| Kessleria inexpectata | TLMF Lep 03126 | PHLAD141-11 | BOLD:ABZ6230 | 658[0n] | 4 |
| Kessleria insubrica | TLMF Lep 01852 | PHLAB1052-10 | BOLD:AAP0590 | 658[0n] | 2 |
| Kessleria insubrica | TLMF Lep 03110 | PHLAD125-11 | BOLD:AAP0590 | 658[0n] | 4 |
| Kessleria insubrica | TLMF Lep 03109 | PHLAD124-11 | BOLD:AAP0590 | 658[0n] | 4 |
| Kessleria insubrica | TLMF Lep 01853 | PHLAB1053-10 | BOLD:AAP0590 | 658[0n] | 4 |
| Kessleria klimeschi | TLMF Lep 01701 | PHLAB901-10 | BOLD:AAO4098 | 658[0n] | 2 |
| Kessleria klimeschi | TLMF Lep 03129 | PHLAD144-11 | BOLD:AAO4098 | 658[0n] | 4 |
| Kessleria klimeschi | TLMF Lep 01702 | PHLAB902-10 | BOLD:AAO4098 | 658[0n] | 2 |
| Kessleria klimeschi | TLMF Lep 01863 | PHLAB1063-10 | BOLD:AAO4098 | 658[0n] | 4 |
| Kessleria klimeschi | TLMF Lep 03128 | PHLAD143-11 | BOLD:AAO4098 | 658[0n] | 4 |
| Kessleria nivescens | TLMF Lep 03114 | PHLAD129-11 | BOLD:ACF5424 | 658[0n] | 4 |
| Kessleria nivescens | TLMF Lep 03112 | PHLAD127-11 | BOLD:AAO9371 | 658[0n] | 4 |
| Kessleria nivescens | TLMF Lep 03117 | PHLAD132-11 | BOLD:ABY5964 | 658[0n] | 4 |
| Kessleria nivescens | TLMF Lep 01854 | PHLAB1054-10 | BOLD:AAO9371 | 658[0n] | 4 |
| Kessleria nivescens | TLMF Lep 03111 | PHLAD126-11 | BOLD:AAO9371 | 658[0n] | 4 |
| Kessleria nivescens | TLMF Lep 01856 | PHLAB1056-10 | BOLD:ACF5424 | 658[0n] | 2 |
| Kessleria nivescens | TLMF Lep 01855 | PHLAB1055-10 | BOLD:AAO9371 | 658[0n] | 2 |
| Kessleria nivescens | TLMF Lep 03115 | PHLAD130-11 | BOLD:ACF5424 | 658[0n] | 4 |
| Kessleria nivescens | TLMF Lep 11058 | LEATB881-13 | BOLD:ACF5424 | 658[0n] | 2 |
| Kessleria nivescens | TLMF Lep 11655 | LEATE243-13 | BOLD:ACF5424 | 658[0n] | 2 |
| Kessleria nivescens | TLMF Lep 03116 | PHLAD131-11 | BOLD:ACF5424 | 658[0n] | 4 |
| Kessleria nivescens | TLMF Lep 11059 | LEATB882-13 | BOLD:ACF5424 | 658[0n] | 2 |
| Kessleria nivescens | TLMF Lep 03113 | PHLAD128-11 | BOLD:ACF5424 | 658[0n] | 4 |
| Kessleria nivescens | TLMF Lep 11656 | LEATE244-13 | BOLD:ACF5424 | 658[0n] | 2 |
| Kessleria orobiae | TLMF Lep 03175 | PHLAD190-11 | BOLD:AAV6685 | 658[0n] | 4 |
| Kessleria orobiae | TLMF Lep 09971 | PHLAW174-13 | BOLD:AAV6685 | 658[0n] | 2 |
| Kessleria orobiae | TLMF Lep 09973 | PHLAW176-13 | BOLD:AAV6685 | 658[0n] | 2 |
| Kessleria orobiae | TLMF Lep 09972 | PHLAW175-13 | BOLD:AAV6685 | 658[0n] | 2 |
| Kessleria orobiae | TLMF Lep 03130 | PHLAD145-11 | BOLD:AAV6685 | 658[0n] | 4 |
| Kessleria petrobiella | TLMF Lep 01861 | PHLAB1061-10 | BOLD:AAP0589 | 658[0n] | 4 |
| Kessleria petrobiella | TLMF Lep 03122 | PHLAD137-11 | BOLD:AAP0589 | 658[0n] | 4 |
| Kessleria petrobiella | TLMF Lep 03123 | PHLAD138-11 | BOLD:AAP0589 | 658[0n] | 4 |
| Kessleria petrobiella | TLMF Lep 01862 | PHLAB1062-10 | BOLD:AAP0589 | 658[0n] | 2 |
| Kessleria pyrenaea | TLMF Lep 08933 | PHLAI438-13 | BOLD:ACE2050 | 658[0n] | 2 |
| Kessleria saxifragae | TLMF Lep 01876 | PHLAB1076-10 | BOLD:AAI8035 | 658[0n] | 4 |
| Kessleria saxifragae | TLMF Lep 00654 | PHLAA614-09 | BOLD:AAI8035 | 647[1n] | 2 |
| Kessleria saxifragae | KLM Lep 00538 | PHLAI063-12 | BOLD:AAI8035 | 627[1n] | 2 |
| Kessleria saxifragae | TLMF Lep 08750 | PHLAH946-12 | BOLD:AAI8035 | 658[0n] | 2 |
| Kessleria saxifragae | TLMF Lep 03152 | PHLAD167-11 | BOLD:AAI8035 | 658[0n] | 4 |
| Kessleria saxifragae | KLM Lep 01119 | PHLAI929-13 | BOLD:AAI8035 | 631[0n] | 2 |
| Kessleria saxifragae | TLMF Lep 11077 | LEATB900-13 | BOLD:AAI8035 | 658[0n] | 2 |
| Kessleria saxifragae | TLMF Lep 08924 | PHLAI429-13 | BOLD:AAI8035 | 658[0n] | 2 |
| Kessleria saxifragae | TLMF Lep 08302 | PHLAH483-12 | BOLD:AAI8035 | 658[0n] | 2 |
| Kessleria saxifragae | TLMF Lep 04211 | PHLAD846-11 | BOLD:AAI8035 | 658[0n] | 2 |
| Kessleria saxifragae | TLMF Lep 03136 | PHLAD151-11 | BOLD:AAI8035 | 658[0n] | 4 |
| Kessleria saxifragae | TLMF Lep 03137 | PHLAD152-11 | BOLD:AAI8035 | 658[0n] | 4 |
| Kessleria saxifragae | TLMF Lep 03143 | PHLAD158-11 | BOLD:AAI8035 | 658[0n] | 4 |
| Kessleria saxifragae | TLMF Lep 03135 | PHLAD150-11 | BOLD:AAI8035 | 658[0n] | 4 |
| Kessleria saxifragae | TLMF Lep 00786 | PHLAA746-09 | BOLD:AAI8035 | 593[0n] | 3 |
| Kessleria saxifragae | TLMF Lep 03138 | PHLAD153-11 | BOLD:AAI8035 | 658[0n] | 4 |
| Kessleria saxifragae | KLM Lep 01118 | PHLAI928-13 | BOLD:AAI8035 | 658[0n] | 2 |
| Kessleria saxifragae | TLMF Lep 08301 | PHLAH482-12 | BOLD:AAI8035 | 658[0n] | 2 |
| Kessleria saxifragae | TLMF Lep 03044 | PHLAD059-11 | BOLD:AAI8035 | 658[0n] | 2 |
| Kessleria saxifragae | TLMF Lep 02903 | PHLAC868-10 | BOLD:AAI8035 | 658[0n] | 2 |
| Kessleria wehrlii | TLMF Lep 03104 | PHLAD119-11 | BOLD:ABZ3416 | 658[0n] | 4 |
| Kessleria wehrlii | TLMF Lep 03105 | PHLAD120-11 | BOLD:ABZ3416 | 658[0n] | 4 |
| Kessleria wehrlii | TLMF Lep 01857 | PHLAB1057-10 | BOLD:ABZ3416 | 658[0n] | 4 |
| Kessleria wehrlii | TLMF Lep 01858 | PHLAB1058-10 | BOLD:ABZ3416 | 658[0n] | 4 |
| Kessleria zimmermanni | TLMF Lep 08922 | PHLAI427-13 | BOLD:ACE2674 | 658[0n] | 2 |
| Kessleria zimmermanni | TLMF Lep 08921 | PHLAI426-13 | BOLD:ACE2674 | 658[0n] | 2 |
| Kessleria zimmermanni | TLMF Lep 08913 | PHLAI418-13 | BOLD:ACE2674 | 658[0n] | 2 |
